# A rapid colorimetric LAMP assay for detection of *Rhizoctonia solani* AG-1 IA causing sheath blight of rice

**DOI:** 10.1038/s41598-020-79117-0

**Published:** 2020-12-16

**Authors:** Prassan Choudhary, Pallavi Rai, Jagriti Yadav, Shaloo Verma, Hillol Chakdar, Sanjay Kumar Goswami, Alok Kumar Srivastava, Prem Lal Kashyap, Anil Kumar Saxena

**Affiliations:** 1 ICAR-National Bureau of Agriculturally Important Microorganisms (NBAIM), Kushmaur, Maunath Bhanjan, Uttar Pradesh 275103 India; 2 ICAR-Indian Institute of Sugarcane Research, Lucknow, 226002 India; 3 ICAR-Indian Institute of Wheat and Barley Research (IIWBR), Karnal, Haryana 132001 India

**Keywords:** Microbiology, Molecular biology, Pathogenesis

## Abstract

*Rhizoctonia solani* is one of the most devastating pathogens. *R. solani* AG-1 IA causes sheath blight in rice, maize, and other Gramineous plants. Accurate identification is essential for the effective management of this pathogen. In the present study, a set of four primers were designed viz. RSPG1, RSPG2, RSPG4, and RSPG5 for polygalacturonase (PG) gene, an important virulence factor in phytopathogenic fungi. All four primer sets showed specific amplification of 300 bp (RSPG1F/R), 375 bp (RSPG2F/R), 500 bp (RSPG4F/R) and 336 bp (RSPG5F/R) amplicons. q-PCR detection using each primer sets could detect up to 10 pg of DNA. We also designed six primers (RS_pg_F3_1/RS_pg_B3_1, RS_pg_FIP_1.1/RS-pg_BIP_1.1, and RS_pg_LF_1/RS_pg_LB_1) for PG gene. Further, a colorimetric LAMP assay developed yielded visual confirmation of the pathogen within 45 min of sample collection when coupled with rapid high throughput template preparation method (rHTTP) from infected samples. The sensitivity of the LAMP assay was as low as 1.65 fg/µl of template DNA and could effectively detect *R. solani* AG-1 IA from diseased plant tissues and soil samples. The LAMP assay was highly specific for *R. solani* as it did not show any amplification with other AG groups of *R. solani* and closely related fungal and bacterial outgroups. This study will help in designing an effective point of care diagnostic method for early monitoring of *R. solani* and thereby planning timely preventive measures against the pathogen.

## Introduction

Rice is considered as one of the most important cereal crops in the world, especially in the South Asian countries^1^. Globally, rice production suffers from various diseases and rice sheath blight is one of the major disease among them. *Rhizoctonia solani* is a ubiquitous pathogen of rice, vegetables^2^, grains^3^, grasses^4^, etc. causing sheath blight which is the second most devastating disease in rice^5,6^. *R. solani* results in considerable yield losses (10–25%) in many crops. This fungal pathogen is known to produce a number of virulence factors which include toxins and hydrolytic enzymes involved in degradation of cell walls^7–10^. Cultivation of high-yielding, semi-dwarf rice varieties requiring application of higher doses of nitrogenous fertilizers have intensified the global occurance of sheath blight. Favourable environment and cultivation of susceptible rice varieties may result in loss of yield as high as 50%^11^. It has been reported that losses due to sheath blight disease alone in India has been up to 54.3%^12^.

Availability of simple and specific diagnostic assays form the base of ecological and epidemiological studies of plant pathogens. Detection of a particular pathogen in the crop or the soil, determining the threshold levels of inoculum provides necessary information on which disease management strategies can be framed. Continuous efforts are being made to develop simple, reliable rapid methods for disease diagnosis and pathogen identification. Identification strategies based on culture and morphology charters are time consuming and require significant taxonomic expertise. Mazzola et al. made initial attempts to design molecular markers for the identification of *R. oryzae*^13^. Lees et al. developed specific and sensitive PCR assay for detection and quantification of *R. solani* AG-3 causing stem canker and black scurf of potato^14^. Woodhall et al. also reported specific quantitative real time PCR assay for detection of *R. solani* AG3-PT which is an important pathogen of potato^15^. Loop mediated isothermal amplification (LAMP), a visual detection method, works on the strand DNA displacement of the target DNA and is being used as a reliable method for the detection of pathogens and plant disease diagnostics. There are few reports on development of LAMP assays for detection of *R. solani* and *R. zeae* causing soybean seedling blight and charcoal rot. Majority of these reports on specific detection of *R. solani* have employed rDNA internal transcribed spacer (ITS) region. Apart from ITS regions, other genomic regions or genes like *noxB*, calmodulin, CYP51C, etc. could also be used for specific detection of fungal pathogens^18–20^.

Polygalacturonase (PG) enzymes are members of glycosyl hydrolase family 28 and catalyze the random hydrolysis of 1,4-α-d-galacturosiduronic linkages in pectate and other galacturonans. It is an important virulence factor in many phytopathogenic fungi^21–24^. This enzyme has been exploited as a marker for grouping and detection of *R. solani*. MacNish et al. used pectic zymograms for characterization of *R. solani* AG-8 isolates^25^. Pectic zymogram variations have also been used for characterization and grouping of *R. solani* AG-4 infecting beans and *R. solani* AG 1 isolates infecting tobacco^26,27^. Despite its association with virulence and prior use for fungal detection, no molecular marker is available for this important enzyme. Development of diagnostic marker using such genes can be very useful to monitor virulent isolates of *R. solani* which can help in its effective and timely management. To the best of our knowledge, very little effort has been made to develop diagnostic markers for *R. solani* AG-1 IA isolates mainly infecting rice.

In the present study, we developed a polygalacturonase gene based diagnostic PCR assay for detection of *R. solani* AG1 IA isolates infecting rice and validated its utility in disease detection in infected samples. Recently, our group reported a simple protocol (Rapid high throughput template preparation method: rHTTP method) to prepare PCR ready templates for a wide range of plants^28^ which could be coupled to molecular diagnostics. Therefore, to further develop a simple strategy for on-field detection of the pathogen, a LAMP based assay (coupled with rHTTP method) was designed and compared to the conventional PCR based diagnostics.

## Materials and methods

### Field sampling

#### For fungal isolation

Infected leaf samples (rice and maize) were collected from Punjab and Uttar Pradesh (Table 1). All samples were collected in polypropylene bags and stored at 4 °C prior to use.

**Table 1:** Details of all the purified isolates along with the sampling details, morphology of the cultures on PDA and submission details of the isolates.

S. no	Fungus	NAIMCC accession numbers	Host	Plate images
1	*R. solani* isolate PU RS1	NAIMCC-F-03220	Rice	
2	*R. solani* isolate PU RS2	NAIMCC-F-03221	Rice	
3	*R. solani* isolate PU RS3	NAIMCC-F-03222	Rice	
4	*R. solani* isolate PU RS4	NAIMCC-F-03223	Rice	
5	*R. solani* isolate PU RS5	NAIMCC-F-03224	Rice	
6	*R. solani* isolate PU RS6	NAIMCC-F-03225	Rice	
7	*R. solani* isolate MRS 1	NAIMCC-F-03226	Maize	
8	*R. solani* isolate MRS 2	NAIMCC-F-03227	Maize	
9	*R. solani* isolate MRS 3	NAIMCC-F-03228	Maize	
10	*R. solani* isolate MRS 4	NAIMCC-F-03229	Maize	
11	*R. solani* isolate MRS 5	NAIMCC-F-03230	Maize	
12	*R. solani* isolate RS14	NAIMCC-F-03231	Rice	
13	*R. solani* isolate RS15	NAIMCC-F-03232	Rice	
14	*R. solani* isolate RS16	NAIMCC-F-03233	Rice	
15	*R. solani* isolate RS17	NAIMCC-F-03234	Rice	
16	*R. solani* isolate RS18	NAIMCC-F-03235	Rice	
17	*R. solani* isolate RS19	NAIMCC-F-03236	Rice	
18	*R. solani* isolate RSDSR 1	NAIMCC-F-03237	Rice	
19	*R. solani* isolate RSDSR 2	NAIMCC-F-03238	Rice	
20	*R. solani* isolate RSDSR 3	NAIMCC-F-03239	Rice	
21	*R. solani* isolate RSDSR 4	NAIMCC-F-03240	Rice	
22	*R. solani* isolate RSDSR 5	NAIMCC-F-03241	Rice	
23	*R. solani* isolate RSDSR 6	NAIMCC-F-03242	Rice	
24	*R. solani* isolate RSDSR 7	NAIMCC-F-03243	Rice	
25	*R. solani* isolate RSDSR 8	NAIMCC-F-03244	Rice	
26	*R. solani* isolate RSDSR 9	NAIMCC-F-03245	Rice	
27	*R. solani* isolate RSDSR 10	NAIMCC-F-03246	Rice	
28	*R. solani* isolate RSDSR 11	NAIMCC-F-03247	Rice	
29	*R. solani* isolate RSDSR 12	NAIMCC-F-03248	Rice	
30	*R. solani* isolate RSDSR 13	NAIMCC-F-03249	Rice	
31	*R. solani* isolate RSDVS 1	NAIMCC-F-03250	Rice	
32	*R. solani* isolate RSKVN 1	NAIMCC-F-03251	Rice	
33	*R. solani* isolate RSHVN 1	NAIMCC-F-03252	Rice	
34	*R. solani* isolate RSMIR 1	NAIMCC-F-03253	Rice	
35	*R. solani* isolate RS36	NAIMCC-F-03294	Rice	
36	*R. solani* isolate RS37	NAIMCC-F-03295	Rice	
37	*R. solani* isolate RS38	NAIMCC-F-03296	Rice	
38	*R. solani* isolate RS39	NAIMCC-F-03297	Rice	
39	*R. solani* isolate RS40	NAIMCC-F-03298	Rice	
40	*R. solani* isolate RS41	NAIMCC-F-03299	Rice	
41	*R. solani* isolate RS42	NAIMCC-F-03300	Rice	
42	*R. solani* isolate RS43	NAIMCC-F-03301	Rice	
43	*R. solani* isolate RS44	NAIMCC-F-03302	Rice	
44	*R. solani* isolate RS45	NAIMCC-F-03303	Rice	
45	*R. solani* isolate RS46	NAIMCC-F-03304	Rice	
46	*R. solani* isolate RS47	NAIMCC-F-03305	Rice	
47	*R. solani* isolate RS48	NAIMCC-F-03306	Rice	
48	*R. solani* isolate RS49	NAIMCC-F-03307	Rice	
49	*R. solani* isolate RS50	NAIMCC-F-03308	Rice	
50	*R. solani* isolate RS51	NAIMCC-F-03309	Rice	
51	*R. solani* isolate AG1L1	NAIMCC-F-03039	Rice	

#### For marker validation

Infected and healthy rice leaf samples were collected from fields of ICAR- Indian Institute of Soil Science, Mau, Uttar Pradesh and Uttar Banga Krishi Viswavidyalaya, Cooch Behar, West Bengal (India).

### Isolation and morphological identification of *R. solani* AG-1 IA isolates from diseased samples

Infected tissue parts were cut (~ 1 cm), sterilized in 1% (w/v) sodium hypochlorite solution and placed on water agar for initial isolation. The pure cultures were characterized morphologically based on their appearance and sclerotial features.

### Development and validation of diagnostic markers

#### Primer designing and conventional PCR assay optimization

Available nucleotide sequences of polygalacturonase gene (HQ197936, HQ197944, FJ544456, KP896519, KP896521, KP896522) of *R. solani* AG-1 IA were downloaded from NCBI database and aligned with ClustalW in MEGA 7.0^29^. From the aligned sequences, conserved regions were identified. Four sets of forward and reverse primers were picked manually from these conserved regions (Table 2). Self complimentary and hairpin formation for the primer sets were checked using OligoCalc program^30^. Specificity of the primer sets were checked using primer BLAST tool (https://www.ncbi.nlm.nih.gov/tools/primer-blast/index.cgi) available at National Center for Biological Information (NCBI). Annealing temperatures for the primer sets were determined using gradient PCR. PCR assays were performed in a total volume of 25 μl by mixing 10 × PCR buffer (2.5 μl), dNTPs (1.5 μl, 50 µM each), 1 μl (10 mM) each of forward and reverse primer, 1.0 U of Taq DNA polymerase (Genei, Bangalore, India), and 2 µl of template DNA (~ 50 ng). PCR was performed in a G Storm GS4 thermal cycler (Somerset, UK) with initial denaturation at 95 °C for 5 min followed by 35 cycles of denaturation at 95 °C for 1 min, annealing for 1 min (RSPG1F/R, 2F/R and 4F/R—52.4 °C, RSPG5F/R—54 °C) (Here we defined these four primer sets as RSPG q-PCR primer sets), extension at 72 °C for 1 min and the final extension of 72 °C for 10 min. PCR products were electrophoresed on 1.5% agarose gel and the photographs were documented in a gel documentation system (Universal Hood 2, BioRad, USA). PCR products (amplified with RSPG2F/R) of 14 random isolates were purified using Wizard SV Gel and PCR Clean up System (Promega, USA) following manufacturers protocol and sequenced from Eurofins Scientific, India. Identity of the sequences was verified through performing BLAST search at NCBI (Supplementary Table S1). The sequences were deposited in NCBI Genbank as accession numbers MT882056- MT882069.

**Table 2:** Primers designed for conventional PCR and q-PCR.

S. No	Primer name	Forward primer/Reverse primer	Tm	GC%	Gradient temp (°C)	Annealing temp (°C)
1	RSPG1F	GGAGACGTAAAGTTCGGAGTTG	60.3	50	52–62	52.4
	RSPG1R	AGGGTTCGAGATGCTGTAGGTA	60.3	50		
2	RSPG2F	TGCAAACCTTACCTCTGCTACA	58.4	45.5	52–62	52.4
	RSPG2R	ATCCATCTGCATTCTTAGGTGG	58.4	45.5		
3	RSPG4F	GTTAGAATCAAGACCTTTGCGG	58.4	45.5	52–62	52.4
	RSPG4R	CGGTGGTGCAGTTGAAGAG	58.8	57.9		
4	RSPG5F	ATTTCGGCACCTTGAATA	56.5	40.9	52–62	54
	RSPG5R	TGAATGCGTGAATGTTCT	56.5	40.9		

#### Microbial cultures for validation

Fungal and bacterial cultures procured from various sources (Table 3) were used for validation of PCR assay.

**Table 3:** Various bacterial and fungal cultures used as references for assay validations.

S. no	Fungal outgroups	Obtained from	Used for
1	*Colletotrichum capsici* isolate CABI-063597	NAIMCC-F-00638	PCR, LAMP
2	*Sclerotium. rolfsii*	NAIMCC-F-03053	LAMP
3	*Sclerotinia sclerotiorum* isolate AS1	NAIMCC-F-03341	PCR
4	*Trichoderma asperellum* isolate P2	NAIMCC-F-03330	LAMP
5	*Fusarium oxyporum* f. sp. *lycopersici*	NAIMCC-F-00889	PCR, LAMP
6	*Curvularia prasadii* isolate CABI-284252	NAIMCC-F-00704	LAMP
7	*Cochiliobolus tuberculatus* isolate CABI-043707	NAIMCC-F-00625	LAMP
8	*Alternaria alternata* isolate CABI-359781	NAIMCC-F-00067	PCR, LAMP
9	*Ustilaginoidea virens* isolate UV2	NAIMCC-F-02995	LAMP
10	*Sarocladium oryzae*	NAIMCC-F-01633	LAMP
11	*Curvularia oryzae* isolate CABI-160069	NAIMCC-F-00699	LAMP
12	*Curvularia lunata* isolate RHS/T556	NAIMCC-F-02904	LAMP
13	*Mangaporthe oryzae* isolate MG1	Dr. N. Sahana, UBKV, West Bengal, India	LAMP
14	*Rhizoctonia oryzae-sativae* isolate MV1	MTCC-9666	LAMP
15	*Fusarium fujikuroi* isolate RPF19	NAIMCC-F-03979	LAMP
16	*Trichoderma viride* isolate OIPP 8315	NAIMCC-F-03110	PCR
17	*R. solani* AG-3 isolate 1	MTCC 4633	LAMP
18	*R. solani* AG-3 isolate 3	MTCC 4634	LAMP
19	*R. solani* AG-7	MTCC 2162	LAMP
20	*R. solani* AG-1 IB isolate M2	Dr. SK Goswami, ICAR-Indian Institute of Sugarcane Research, Lucknow, India	LAMP
21	*R. solani* AG 2-2IIIB isolate O1	Dr. SK Goswami, ICAR-Indian Institute of Sugarcane Research, Lucknow, India	LAMP
22	*R. solani* AG-8 isolate S1	Dr. SK Goswami, ICAR-Indian Institute of Sugarcane Research, Lucknow, India	LAMP
23	*Bacillus subtilis* isolate MG1	NAIMCC-B-00116	PCR
24	*Pseudomonas plecoglossicida* isolate S7	NAIMCC-B-00397	PCR, LAMP

#### Molecular detection and sensitivity of the diagnostic markers using q-PCR

Molecular detection and sensitivity of the diagnostic markers were determined by q-PCR (G8830A AreaMx Real-Time PCR, Agilent Technologies, USA) using SYBR green I as fluorescent molecules. Brilliant III Ultra-Fast SYBR® Green QPCR Master Mix was used for the reactions provided by Agilent Technologies, USA. The sensitivity assay for all the primers were performed using the protocol reported by Chakdar et al. (2019) with some modifications^20^. The real time qPCR assay was performed using all four RSPG primer sets (10 pmol/µl. q-PCR conditions were: initial heat activation at 95 °C for 10 min, followed by 40 cycles of amplification by 3 step cycling, denaturation was carried out at 95 °C for 5 s, annealing at (RSPG1F/R, 2F/R and 4F/R—52 °C, RSPG 5F/R—54 °C) for 30 s, and extension at 72 °C for 15 s with a reaction volume of 10 µl. Melting curve analysis was performed by heating the plate at 95 °C for 30 s, and incubating at 65 °C for 30 s, then heating to 95 °C for 30 s. DNA (genomic DNA of *R. solani* isolate PU RS1) intensity and purity was quantified by Nano drop (MicroIndia Co. Ltd). Serial dilutions of 100 ng/µl, 10 ng/µl, 1.0 ng/µl, 0.1 ng/µl and 0.01 ng/µl of DNA was prepared using nuclease free MiliQ water. A reference reaction setup was performed targeting actin gene. A no template control (NTC) was kept for all the experiments designed in order to check the specificity of the reactions. All dilutions of DNA (10–0.01 ng/µl) were used as template for performing the q-PCR experiments. All the experiments were carried out in triplicates. AreaMx V1.7 software was used to analyze the data generated. Efficiency of the q-PCR was calculated by plotting Ct values against –log (DNA concentration in g) and determining the regression equation.

### LAMP assay validation, specificity, and sensitivity

#### LAMP assay standardization

Primers for the LAMP assay were designed using the polygalacturonase gene (NCBI accession number : MT882069) sequenced by using conventional PCR primer sets in this study. Primer Explorer v5 was used to design the primer sets (Eiken Chemical Co., Ltd., Tokyo, Japan) (Table 4). Primer BLAST was done with each primer designed to check the specificity of the newly designed primers. The LAMP system used in this study consisted of 12.5 µl WarmStart Colorimetric LAMP 2X Master Mix (New England Biolabs), 2.5 µl primer mix (RS_pg_F3_1/RS_pg_B3_1, RS_pg_FIP_1.1/RS-pg_BIP_1.1, and RS_pg_LF_1/RS_pg_LB_1) (Here, we defined these six primers as RSPG LAMP primer sets), 3 µl template DNA, and MiliQ water (Promega, USA) for a final volume of 25 µl. The assay conditions were followed according to the manufacturer's protocol (65 °C for 30 min). Visual confirmation was carried out as yellow colour development indicated positive reaction while red colour indicated no reaction. The amplified LAMP products were further observed on 2% agarose gel with EZ-Vision One as DNA dye (Amresco, USA) to confirm amplification (Fig. 4).

**Table 4:** Primers designed for LAMP assay.

Primer name	Sequences (5′–3′)	Type	Length	GC%	T_m_ °C
RS_pg_F3_1	GGCCAAGCCAGTAAGTCTT	Forward outer	19	52.6	55
RS_pg_B3_1	TGAGGTCGGTTGGTTTGC	Backward outer	18	55.6	55.7
RS_pg_FIP_1.1	CCAGTGCCCAGGGAATGTCTAG-TTTT-CTGTTCCCGTACTTCTGCG	Forward inner	22–19	59.1–57.9	59.5–55.7
RS-pg_BIP_1.1	GCACTAATGTGACCCTGCGTGG-TTTT-ACAGCATCCCACCATTGC	Backward inner	22–18	59.1–55.6	60.6–56
RS_pg_LF_1	GGCATACCATTAACCGGTGC	Forward loop forming	20	55	56.5
RS_pg_LB_1	TGGATCGACTCGCACGG	Backward loop forming	17	64.7	57.5

#### Sensitivity of LAMP assay

The sensitivity of the LAMP assay was determined using different *R. solani* AG-1 IA DNA concentrations in descending order by 10- fold serial dilution with sterilized double-distilled water from 165 ng/µl to 1.65 fg/µl. Serially diluted DNA (3 µl each) was used as template DNA in the LAMP reaction to quantify its sensitivity in a thermal cycler at a uniform temperature of 65 °C for 30 min.

#### Specificity of LAMP assay

To check the specificity, fungal and bacterial cultures listed in Table 3 were used. A “no template control” was kept in all experimental set ups and the experiments were repeated three times.

#### In vitro and pot experiments for validation of LAMP assay

Leave sheaths of rice were infected using virulent strains of *R. solani* AG-1 IA both by moist chamber technique and rice plants grown in pots in order to check early detection of this pathogen^34^. In case of a moist chamber, leaves were cut to 2 cm in size, placed on a glass slide and the set up was kept on moist filter paper inside sterile glass petri dishes. Sclerotia was placed on the top of the leaves and the petri dishes were incubated at 28 ± 2 °C. While infecting leaves in a moist chamber, disease severity was kept at three levels (1 sclerotia—+, 2 sclerotia—++, and 3 sclerotia—+++) in order to test the applicability of LAMP assay with different inoculum loads of disease occurance. In case of rice plants in pots, the sclerotia was placed in the leaf sheath and covered with moist cotton to provide appropriate conditions for the infection process. The pots were maintained in net house conditions. All the healthy and infected samples were collected after 24 h to perform the LAMP assay.

#### Validation of LAMP assay using soil DNA

To determine the applicability of the LAMP method, soil samples were collected from rice fields at ICAR-IISS, Maunath Bhanjan, India. From 1 m^2^ area, four samples each weighing 50 g were collected, dried under shade, finely ground using sterile mortar-pestle and sieved. 25 g from each of these four samples were pooled together. Total soil DNA was extracted from the pooled sample in six replications using a commercial kit (FastDNA Spin Kit for Soil, MP Biomedicals, USA) following minor modifications to manufacturer’s protocol. DNA was quantified using Nano Drop and pooled DNA sample was used for the LAMP assay. The time for LAMP assay was optimized and the final assay conditions were 65 °C for 45 min. The rice field was monitored for disease incidence for one month after the collection of soil samples.

### Isolation of genomic DNA

#### Fungal

Genomic DNA from fungal mass of seven days old culture was extracted following the method described by Kumar et al. with minor modifications^31^.

#### Plant

Total genomic DNA from plant material was extracted according to DNA extraction methodologies described by Doyle and Doyle with modifications from the original method^32,33^. Leaf samples were washed and dried with paper towels to eliminate excess dirt. 0.5 g leaf tissue was used for DNA extraction. DNA extracted from infected and healthy leaf samples was used as template for specific detection of *R. solani* AG-1 IA in rice tissue. For all the LAMP assays, rHTTP method was followed for template preparation directly from infected and healthy tissues^28^.

## Results

### Morphological characterization

Fifty-one different *R. solani* AG-1 IA isolates were purified from infected leaf samples collected from Mau, Ghazipur & Varanasi districts of Uttar Pradesh and Punjab. Colour of the colonies of the isolates ranged from pale brown to yellowish brown or whitish brown (Supplementary Table S2) Location of the sclerotia was either central, peripheral or scattered while the colour ranged from light to dark brown. Majority of the isolates had micro sized sclerotia while few had macro sized (Supplementary Table S2). Distinct morphological features of the isolates established their identity as *Rhizoctonia solani* AG-1 IA. All the morphologically identified isolates were deposited in NAIMCC at ICAR-NBAIM, Mau as NAIMCC F-03220-03039 (Table 1).

### Development and validation of diagnostic markers

#### Specificity, sensitivity and validation of diagnostic markers (RSPG) on pure isolates and environmental samples

**Figure 1:**
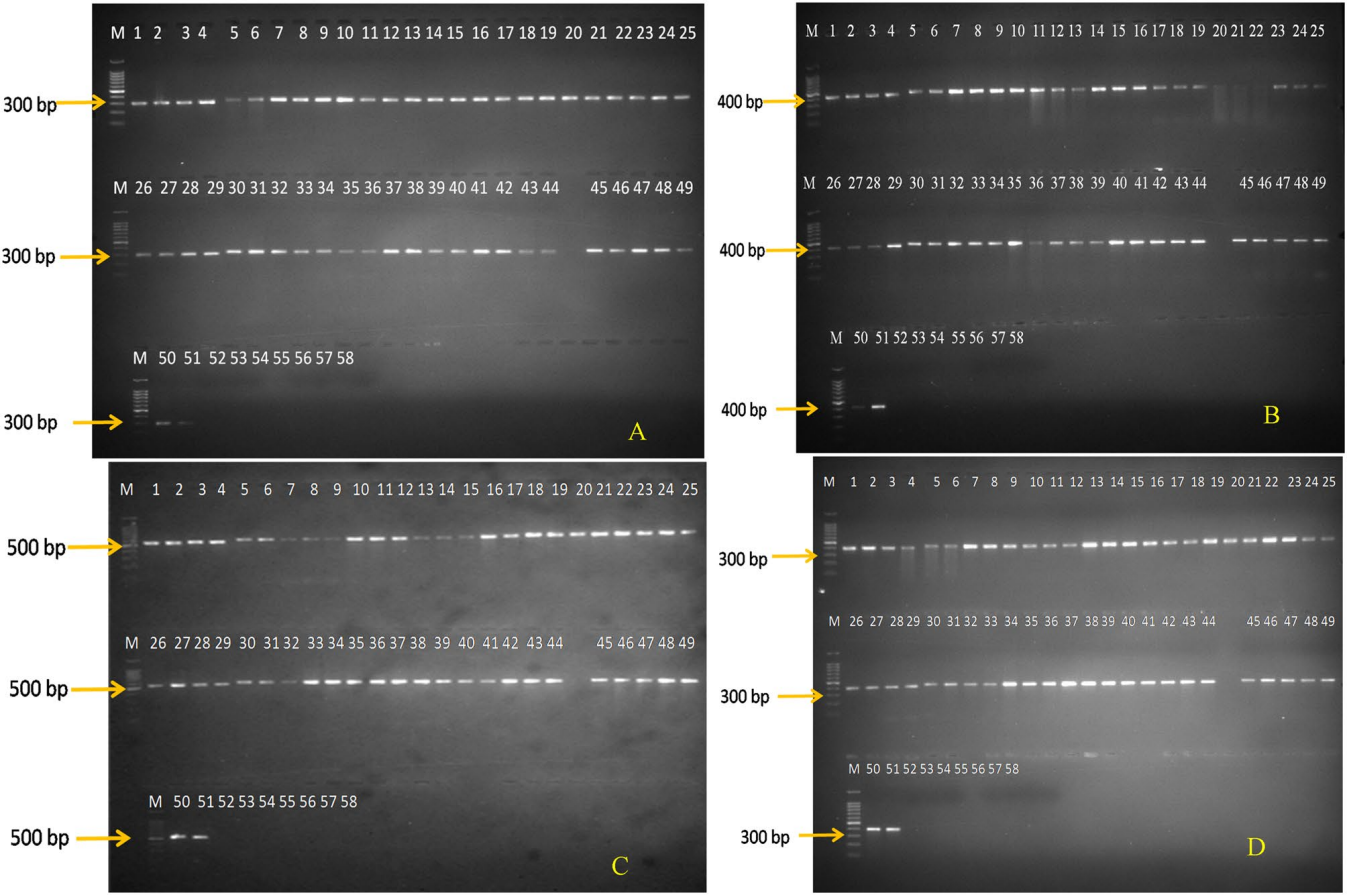
**(A)** PCR amplification using primer pair RSPG1Fand RSPG1R showing amplification product of 300 bp. **(B)** PCR amplification using primer pair RSPG2F and RSPG2R showing amplification product of 375 bp. **(C)** PCR amplification using primer pair RSPG4F and RSPG4R showing amplification product of 500 bp. **(D)** PCR amplification using primer pair RSPG5F and RSPG5R showing amplification product of 336 bp. Lane M is a 100-bp DNA marker. Lanes 1–51 represent different *Rhizoctonia solani* AG-1 IA strains; while lanes 52–58 are *S. sclerotiorum*, *T. viride, F. oxyporum*, *A. alternata*, *C. capsici, B. subtilis,* and *P. plecoglossicida* respectively. All DNA were extracted from fungal isolates.

To evaluate the effectiveness and specificity of RSPG q-PCR primer sets, PCR was performed using purified DNA of the 51 isolates of *R. solani* AG-1 IA which resulted in desired amplification of 300 bp, 375 bp, 500 bp and 336 bp amplicons respectively. No amplification was observed in fungal out groups and bacterial species (*Sclerotium sclerotiorum, Trichoderma viride, Fusarium oxysporum, Alternaria alternata, Colletotrichum capsici, Bacillus subtilis, Pseudomonas plecoglossicida*) used for the PCR specificity assay (Fig. 1A–D). The results confirmed RSPG q-PCR primer sets markers as specific for *R. solani* AG-1 IA*.*

**Figure 2:**
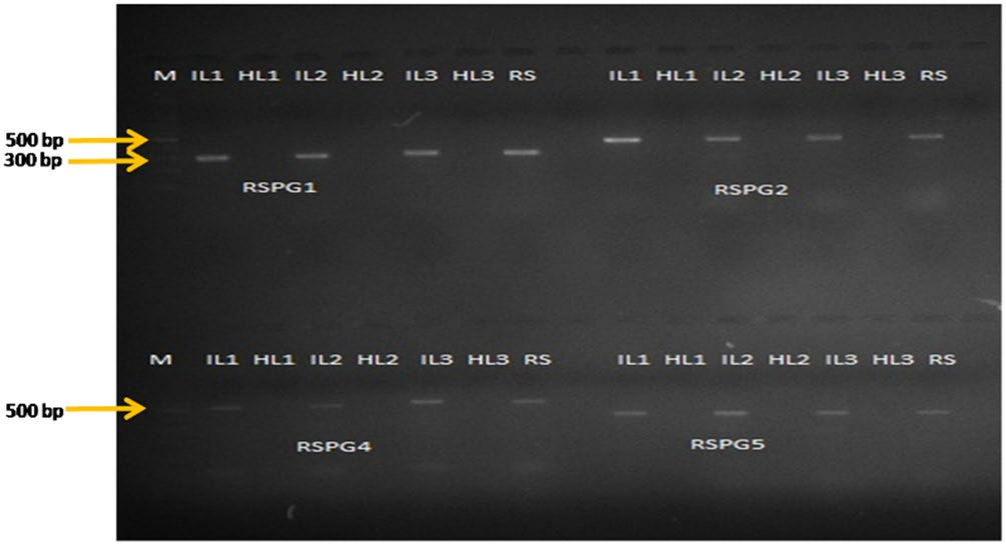
Validation of primer sets of polygalacturonase gene as a diagnostic marker for *R. solani* AG-1 IA isolate AG1L1 (genomic DNA isolated from diseased plant samples). RSPG1 (300 bp), RSPG2 (375 bp), RSPG4 (500 bp) and RSPG5 (336 bp) show distinct amplification bands in case of tissue sheaths infected with *R. solani* AG-1 IA whereas no amplification is observed in healthy tissues. *IL* infected leaf, *HL* healthy leaf, and *RS* *R. solani* strains.

Validation using live infected samples confirmed that the primers designed in this study could clearly detect *R. solani* AG-1 IA from the genomic DNA extracted from infected leaf sheath samples (Fig. 2). No amplification in healthy parts of the plant obtained confirmed the applicability of RSPG primers as diagnostic markers for the specific detection of phytopathogenic *R. solani* AG-1 IA.

#### Molecular detection and sensitivity of diagnostic markers by q-PCR

**Figure 3:**
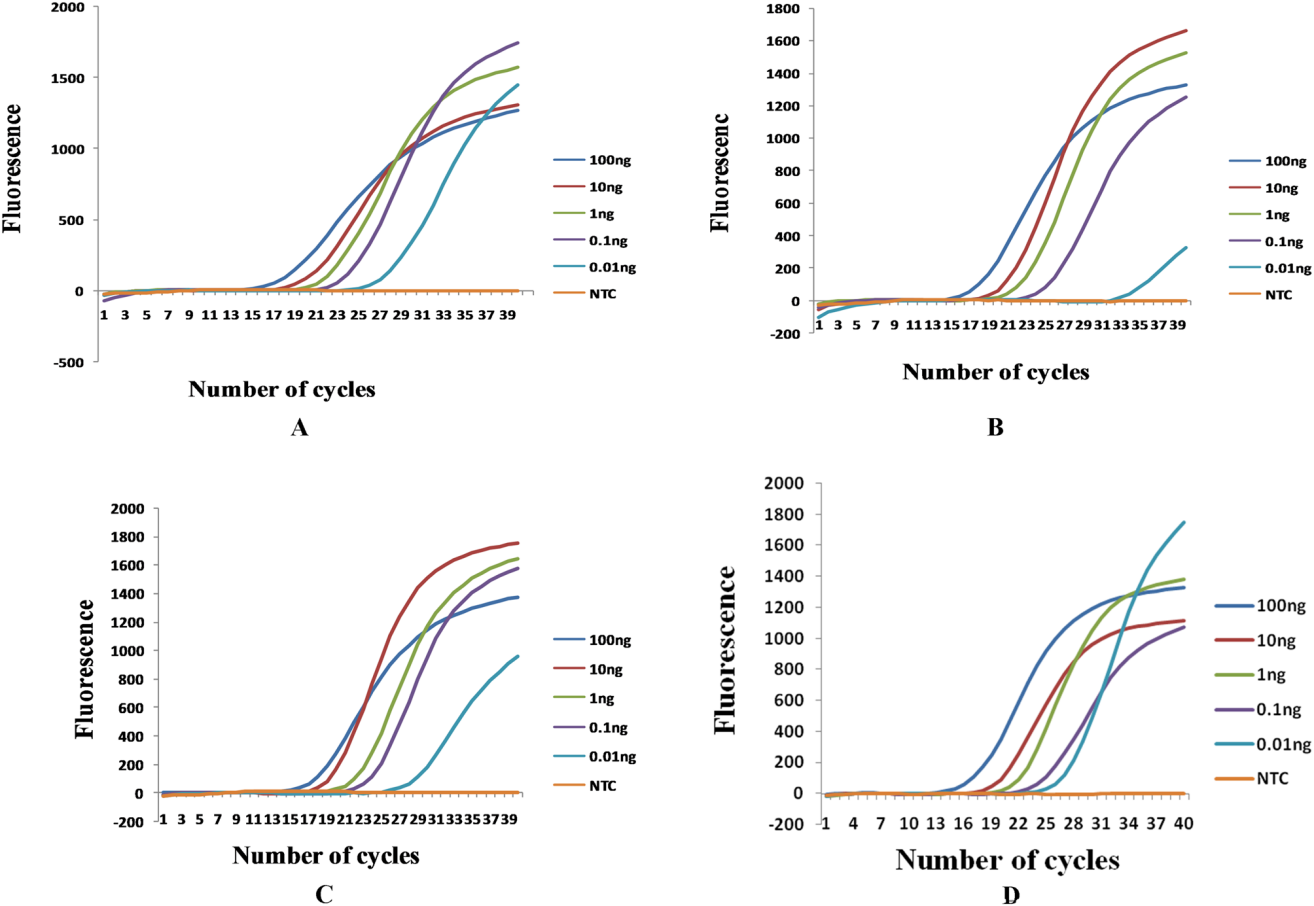
Standard curve for absolute quantification of genomic DNA generated with tenfold serial dilutions of genomic DNA isolated from infected plant material of probable *R. solani* AG-1 IA infection using RSPG primer sets. The curves show the relative fluorescence intensity with respect to the number of PCR cycles. (**A**) RSPG 1 primer set, (**B**) RSPG 2 primer set, (**C**) RSPG 4 primer set, and (**D**) RSPG 5 primer set.

When the DNA extracted from leave sheaths with early symptoms (tiny brown spots) indicating probable *R. solani* infection, was subjected to real time PCR using RSPG q-PCR primer sets, amplification was observed till 0.01 ng/µl (Fig. 3). All the primers could detect 0.01 ng DNA/µl with efficiency ranging from 91 to 97.5% (Supplementary Table S3, File. 5–8). No peaks were observed in no template control (NTC) indicating the specificity of the diagnostic markers. RSPG q-PCR primer sets gave good results with clear fluorescence peaks. The fluorescent peaks corresponding to the amplicons were centered around 84 °C (Supplementary Fig. S1).

#### Validation and specificity of the LAMP assay for R. solani AG-1 IA detection

**Figure 4:**
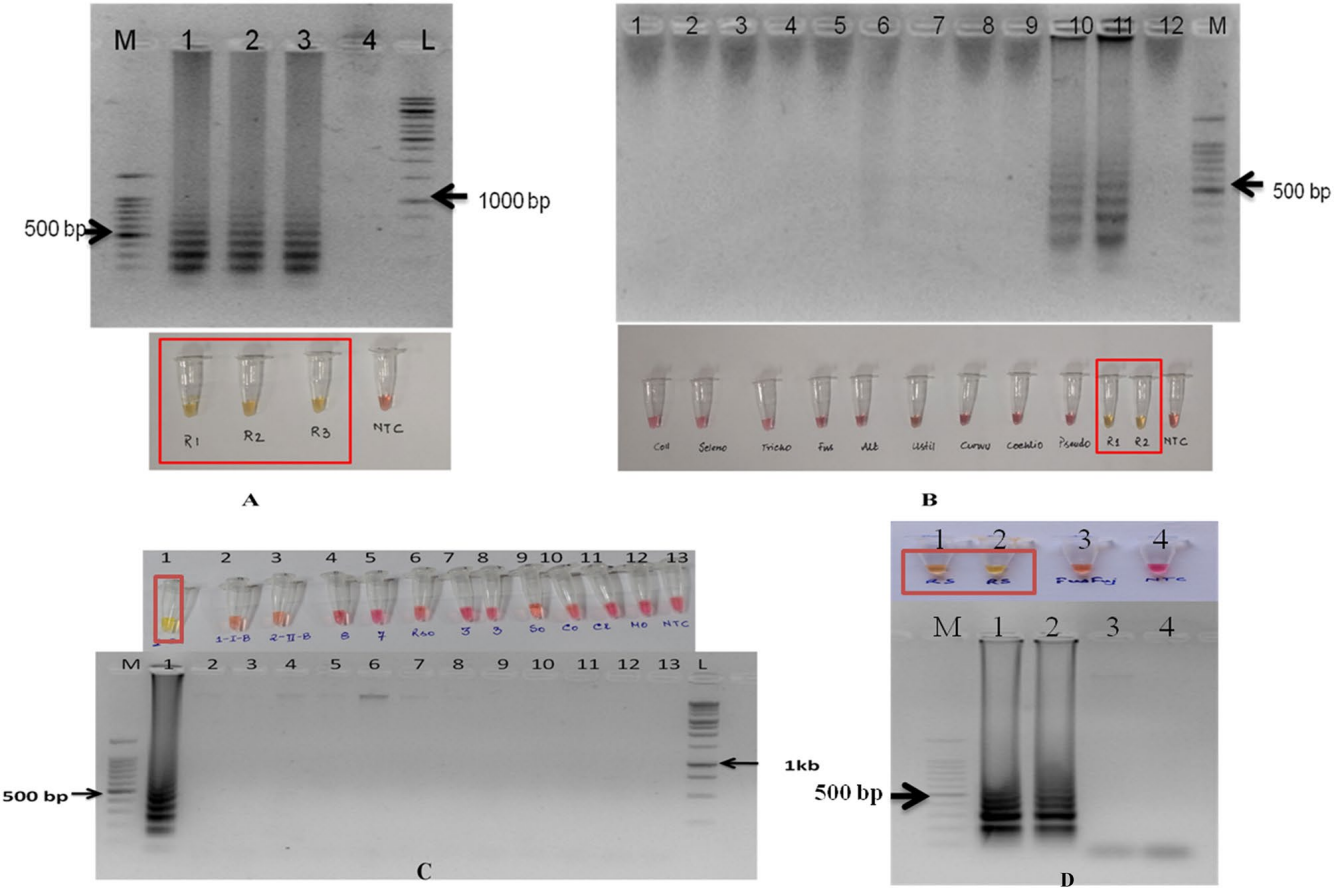
Optimization and validation of LAMP assay. Yellow colour indicated a positive reaction while red/pink colour indicated no reaction. (**A**) LAMP assay optimized with pure fungal isolates with no template control. M: 100 bp (Promega); 1: *R. solani* AG-1 IA isolate PURS1; 2: *R. solani* AG-1 IA isolate PURS2 3: *R. solani* AG-1 IA isolate PURS3; 4: No Template control; L: 1 kb (Generuler). [upper panel: gel photograph; lower panel: colorimetric reactions in PCR tubes] (**B**) Specificity assay of the LAMP assay. L: 1 kb (Generuler); 1: *Colletotrichum capsici* isolate CABI-063597; 2: *Sclerotium rolfsii*; 3: *Trichoderma asperellum;* 4: *Fusarium oxysporum*; 5: *Alternaria alternata*; 6: *Ustilaginoidea virens*; 7: *Curvularia prasadii*; 8: *Cochiliobolus tuberculatus*; 9: *Pseudomonas plecoglossicida* 10: *R. solani* AG-1 IA isolate PURS1; 11: *R. solani* AG-1 IA isolate PURS2; 12: No Template control; M: 100 bp (Promega). [upper panel: gel photograph; lower panel: colorimetric reactions in PCR tubes] (**C**) M: 100 bp (Promega); 1: *R. solani* AG-1 IA isolate PURS1; 2: *R. solani* AG-1 IB 3: *R. solani* AG 2–2 IIIB; 4: *R. solani* AG-8; 5: *R. solani* AG-7; 6: *R. oryzae-sativae* 7: *R. solani* AG-3; 8: *R. solani* AG-3; 9: *Sarocladium oryzae*; 10: *Curvularia oryzae*; 11: *Curvularia lunata*; 12: *Magnaporthe oryzae*; 13: No template control; 14: 1 kb ladder (Promega) [upper panel: colorimetric reactions in PCR tubes; lower panel: gel photograph] (**D**) M: 100 bp (Promega); 1: *R. solani* AG-1 IA isolate PURS1; 2: *R. solani* AG-1 IA isolate PU RS14; 3: *Fusarium fujikuroi* isolate RPF19; 4: No template control [upper panel: colorimetric reactions in PCR tubes; lower panel: gel photograph].

The primer set designed for the study targeted polygalacturonase gene sequenced during the study (Supplementary Fig. S2). The diagnostic marker, once validated, was used for designing the LAMP primers. In silico primer BLAST results for all the primers gave specific hits to *R. solani* AG-1 IA. The *R. solani* AG-1 IA isolates used in the study showed positive reactions with the RSPG LAMP primer sets, but did not amplify in other tested AG groups (AG 1- IB, 2-2IIIB, 3, 7 & 8) and other major rice pathogens (Fig. 4, Supplementary Figs. S3,S4). The LAMP assay and conventional PCR showed the same specificity when validated with other prominent rice pathogens including cross kingdom specificity.

**Figure 5:**
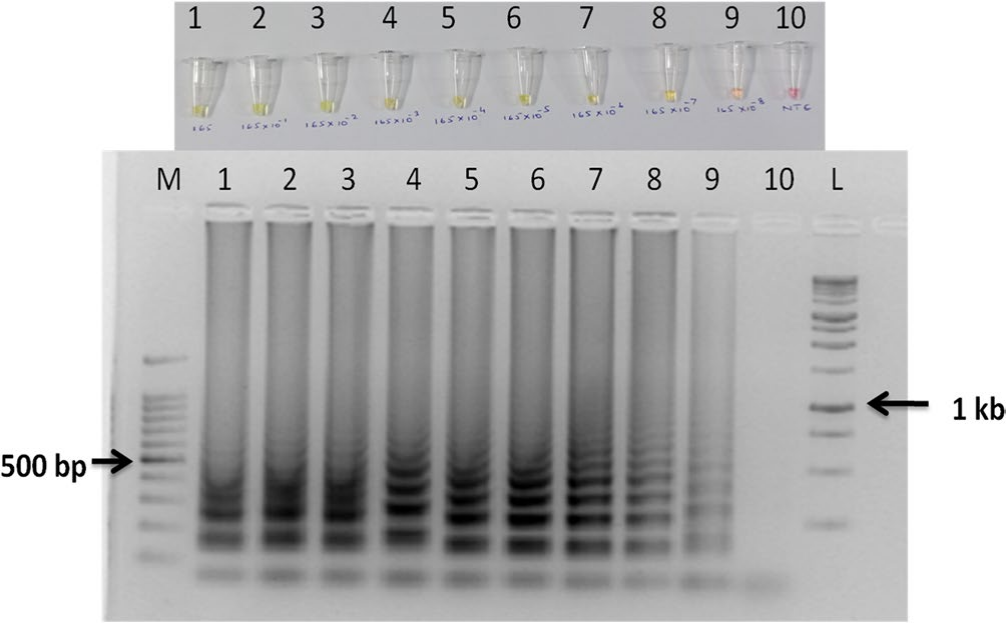
Sensitivity of the LAMP assay when performed with tenfold serial dilution of template DNA. M: 100 bp (Promega); 1: 165 ng/µl; 2: 165 × 10^–1^ ng/µl; 3: 165 × 10^–2^ ng/µl; 4: 165 × 10^–3^ ng/µl; 5: 165 × 10^–4^ ng/µl; 6: 165 × 10^–5^ ng/µl; 7: 165 × 10^–6^ ng/µl; 8: 165 × 10^–7^ ng/µl; 9: 165 × 10^–8^ ng/µl; 10: No template control; L: 1 kb (Promega). Upper panel in the figure shows reaction tubes whereas lower panel shows agarose gel electrophoresis results.

**Figure 6:**
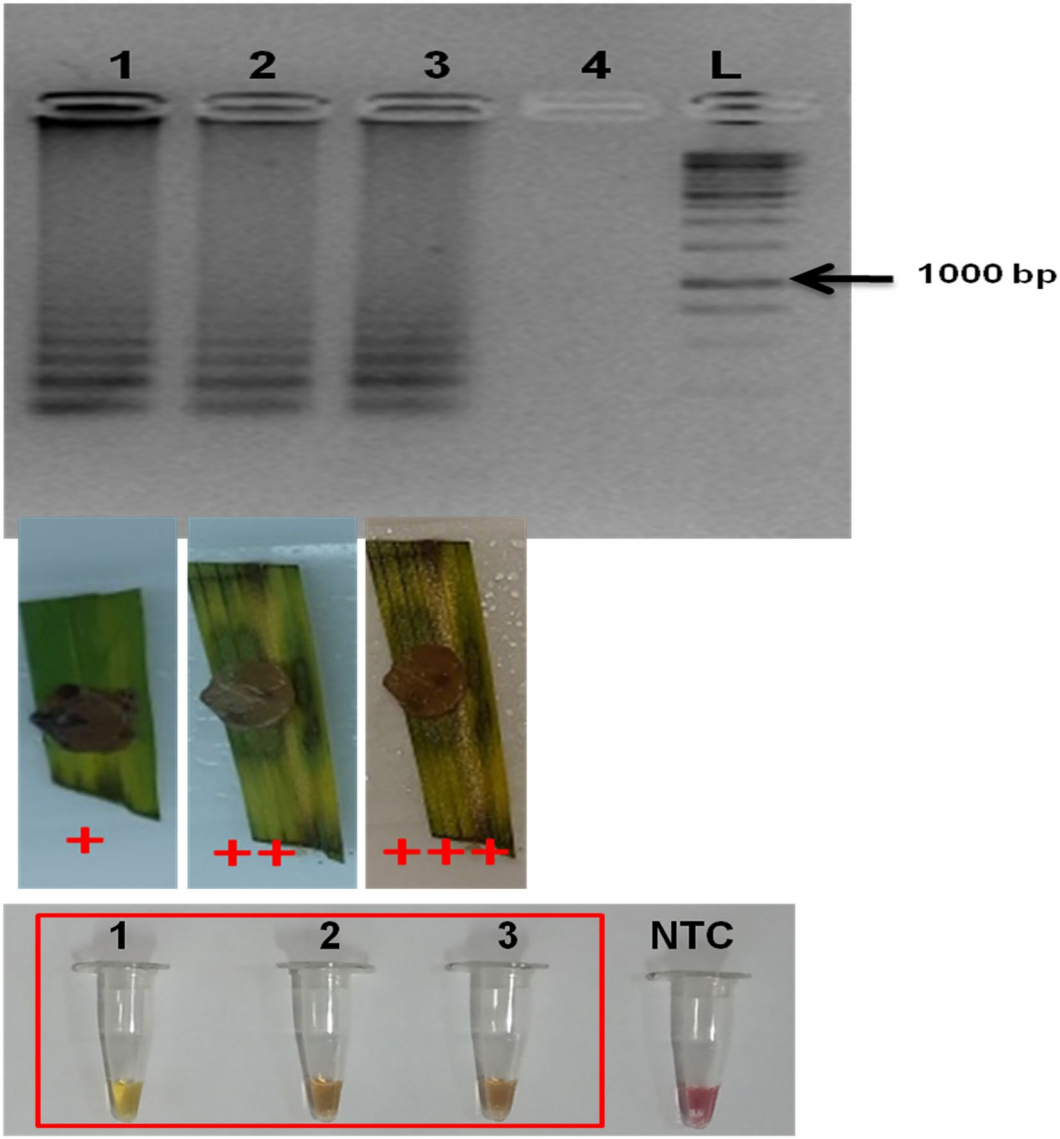
LAMP assay with infected rice leaves of different severity levels (artificial infection). 1: *R. solani* AG-1 IA isolate PURS2 (severity: +); 2: *R. solani* AG-1 IA isolate PURS2 (severity: ++); 3: *R. solani* AG-1 IA isolate PURS2 (severity: +++); 4: No template control; L: 1 kb (Generuler). Upper panel in the figure shows agarose gel electrophoresis results, middle panel shows diseases severity in the samples and lower panel shows reaction tubes.

**Figure 7:**
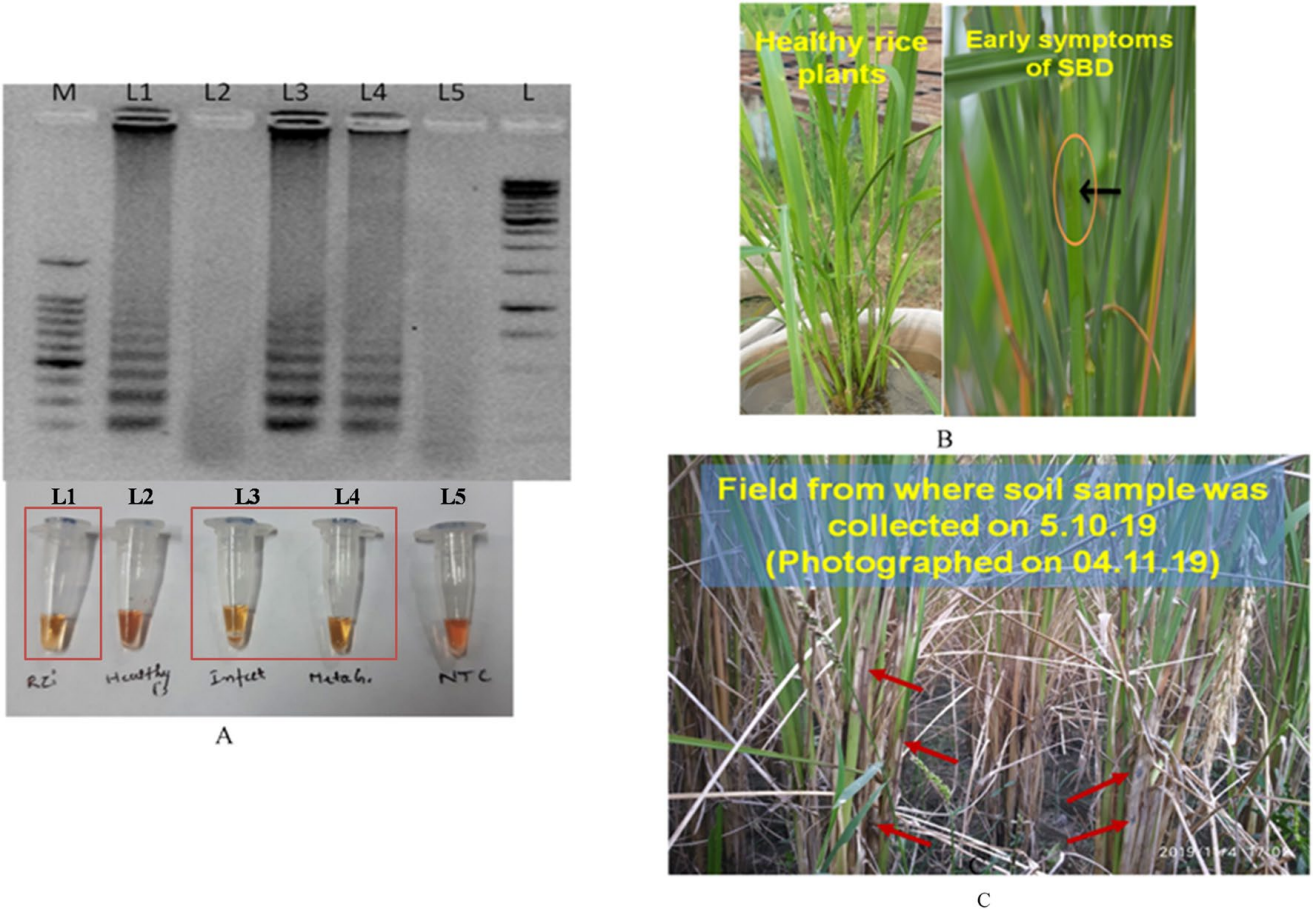
LAMP assay results with environmental samples and soil from rice field. Upper panel in the figure shows agarose gel electrophoresis results whereas lower panel shows reaction tubes. (**A**) M: Marker; L1: *R. solani* AG-1 IA isolate PU RS2; L2: healthy plant; L3: Infected plant; L4: Soil DNA from rice field; L5: NTC. (**B**) Photographs of healthy and early symptoms of sheath blight disease on rice just after 24 h of infection infected rice plants used in the study. Orange color circle indicate lesions (**C**) Disease incidence after one month of collection of soil samples for performing the LAMP assay. Red arrows indicate lesions.

The assay could detect the target gene in 1.65 fg/µl of the template DNA (Fig. 5). rHTTP method was used for the template preparation from diseased plant tissues having sheath blight symptoms. The template was then tested for LAMP assay. Thus, the total time taken for the assay was 45 min (rHTTP: 15 min and LAMP: 30 min). The LAMP assay was also able to detect the pathogen from artificially infected leaf samples with varying degree of disease severity appeared after 24 h of inoculation (Fig. 6). The primer sets were highly specific as it could differentiate between the healthy and infected plant tissue samples showing initial symptoms collected from pot trials just 24 h after the inoculation (Fig. 7). The results obtained highlighted the fact that the LAMP assay developed in this study could efficiently and accurately detect the disease as soon as the pathogen gets entry to the plant.

The assay was further validated using DNA from soil samples from rice fields. The extracted soil DNA samples were quantified (160–180 ng/µl). The LAMP assay gave good results when the assay time was of 45 min for the visual confirmation in the case of soil DNA as template (Fig. 7, Supplementary Fig. S5). The fields from which the soil samples were taken had severe symptoms of Sheath Blight Disease (SBD) when observed after one month of collection of soil samples (Fig. 7c).

## Discussion

Accurate identification of *R. solani* AG-1 IA is essential for its effective management. Excessive and inappropriate use of anti-fungal agents not only hampers the crop production but also damage soil health jeopardizing human health and livestock eventually^35^. SBD of rice hampers the production of rice worldwide ranging from 8 to 50% loss in yield^36^. Many studies are being carried out to increase the resistance of plants against sheath blight but it also involves precise detection of the pathogen^36,37^.

There have been efforts for molecular detection of *R. solani* but no study is reported till date of using a virulence factor as the basis of molecular detection of the pathogen. Polygalacturonase encoding genes form the basis for the infection process of *R. solani* among its various pathotypes^6,38^. This study used PG gene to design four sets of primers and thus the detection is based on virulence factor of the pathogen. The primer sets were tested with the sheath samples collected from farmer’s field where chances of mixed infection remained in a high proportion. Specific bands were seen which detect *R. solani* AG-1 IA among other close relatives of fungal kingdom. These molecular markers can be used for detection of *R. solani* AG-1 IA infection and thus effective use of fungicides can be monitored and will definitely help to devise control strategies for reducing economic losses.

In this study, a total 51 *R. solani* AG-1 IA isolates were obtained from diverse agro-ecological regions of India. An important virulence factor i.e., polygalacturonase gene based diagnostic markers were validated on these isolates of *R. solani* AG-1 IA. PCR based detection of pure cultures of *R. solani* AG-1 IA isolates showed positive amplification while no amplification was obtained for other fungi or bacteria indicating its specificity for *R. solani* AG-1 IA. Further, the designed primer sets could clearly distinguish between healthy and *R. solani* infected leaf samples and even detect the presence of pathogen in samples. Earlier, efforts have been made to detect *R. solani* with distinct pathotypic and genetic diversity. Majority of the studies carried out yet reported no significant relationships between genetic diversity and aggressiveness or geographic origin among populations of *R. solani* AG-1 IA^39,40^. Virulence is independent of molecular variation, and the high levels of genetic recombination may also contribute to new genotypes with a high degree of variation in virulence^41,42^.

As use of PCR based markers is not easy and convenient in the fields, it was required to go for isothermal amplifications which are more convenient to be used in fields. Earlier, Lu et al. reported an ITS gene based LAMP assay to detect *R. solani* causing soybean seedling blight^17^. The assay took more than 2 h and the DNA extraction was tedious and costly which is not suitable for developing point-of-care diagnostics. Patel et al. also targeted ITS region to detect *R. solani* AG-4 in *Dypsis lutescens*, *Fittonia*, *Dianthus* and *Begonia*^16^. Both the studies did not target SBD of rice and targeted the same region of the genome (ITS) for developing the LAMP assay. This study might be the first attempt to develop a LAMP based assay for the detection of *R. solani* AG-1 IA in rice. More importantly, visual confirmation provided by this method and the isothermal amplification property made this method more convenient for pathogen detection. The assay was first optimized using fungal DNA from pure cultures of isolates. In case of detection from live infected tissues (both in vitro and pot trial infections) rHTTP method was employed for template preparation^28^. The integration of rHTTP and LAMP assay approach required a total of only 45 min from sample preparation to pathogen detection. The rHTTP method uses a single temperature during the entire template preparation method, hence the whole experimental set up (right from sample collection to detection) can be performed in any portable water bath or hot plate. LAMP was more simple, cost effective and required less complex instrumentation systems. Moreover, it could detect the pathogen as early as 24 h after the infection (Fig. 7b). Hence, this would be the most appropriate method for on field detection studies. Studies have been reported which have highlighted the use of LAMP based assays for the detection of important phytopathogens like *Rhizoctonia bataticola*^43^, *Talaromyces flavus*^44^, *Alternaria solani*^45^, *Didymella bryoniae*, etc.

Further validation of the assay was done with soil samples collected from a rice field. Results indicated that the optimized assay could successfully detect the presence of pathogen from soil samples although it took a little more time for optimal amplification. The sampling site was marked and kept under observation. As our assay results suggested, we observed devastating symptoms of *R. solani* AG-1 IA infection in the rice plants one month after the sampling date (Fig. 7c). This observation substantiates the potential use of this LAMP based assay for early monitoring sheath blight of rice. Reports on biological control of sheath blight suggest that control measures, if backed up by affordable point of care diagnostic methods as achieved in this study, may be the possible solution for farmers who suffer heavy losses each year due to SBD^46,47^.

## Conclusion

The main features of the developed method are: (1) colorimetric detection, (2) requires less time (45 min), (3) minimum instrumentation required, (4) detection at early stages of the disease, (5) applicable for environmental samples including diseased tissues and soil, (6) coupling with rHTTP further reduces cost, time and effort. With these results in place, we propose this LAMP assay to be used in order to plan effective management strategies so that the damage caused by this phytopathogen can be minimized. Although, the conventional PCR based assays were also quite sensitive but their requirement for instrumentation and lack of visual confirmation make them unsuitable for use in field conditions.

## Supplementary Information

Supplementary Information.

## Data Availability

The datasets generated and analysed during the current study are available from the corresponding author on reasonable request.
