# Supplementary material for: A rapid colorimetric LAMP assay for detection of Rhizoctonia solani AG-1 IA causing sheath blight of rice
Source: Sci Rep. 2020 Dec 16;10:22022. doi: 10.1038/s41598-020-79117-0 (PMC7744555; doi:10.1038/s41598-020-79117-0)
Supplement: Supplementary file 1 — Supplementary Information. [file 41598_2020_79117_MOESM1_ESM.pdf]

A rapid colorimetric LAMP assay for detection of *Rhizoctonia solani* AG-1 IA causing sheath blight of rice

Prassan Choudhary[1], Pallavi Rai[1], Jagriti Yadav[1], Shaloo Verma[1], Hillol Chakdar[1]*, Sanjay Kumar Goswami[1,2], Alok Kumar Srivastava[1], Prem Lal Kashyap[3], Anil Kumar Saxena[1]

[1]ICAR-National Bureau of Agriculturally Important Microorganisms (NBAIM), Kushmaur, Maunath Bhanjan, Uttar Pradesh-275103, India

[2] ICAR-Indian Institute of Sugarcane Research, Lucknow-226002, India

[3] ICAR-Indian Institute of Wheat and Barley Research (IIWBR), Karnal, Haryana-132001

*Corresponding author: hillol.chakdar@gmail.com

Supplementary Table 1. Accession number details of polygalacturonase gene sequenced along with the blast results.

**Strain**	**Accession number**	**Length (bp)**	**BLAST hits**
NAIMCC-F-03237	MT882056	306	*Rhizoctonia solani* AG-1 IA strain YN-7 polygalacturonase 5 (PG5)
NAIMCC-F-03238	MT882057	308	*Rhizoctonia solani* AG-1 IA strain YN-7 polygalacturonase 5 (PG5)
NAIMCC-F-03239	MT882058	313	*Rhizoctonia solani* AG-1 IA strain YN-7 polygalacturonase 5 (PG5)
NAIMCC-F-03241	MT882059	314	*Rhizoctonia solani* AG-1 IA strain YN-7 polygalacturonase 5 (PG5)
NAIMCC-F-03242	MT882060	312	*Rhizoctonia solani* AG-1 IA strain YN-7 polygalacturonase 5 (PG5)
NAIMCC-F-03244	MT882061	319	*Rhizoctonia solani* AG-1 IA strain YN-7 polygalacturonase 5 (PG5)
NAIMCC-F-03247	MT882062	318	*Rhizoctonia solani* AG-1 IA strain YN-7 polygalacturonase 5 (PG5)
NAIMCC-F-03248	MT882063	312	*Rhizoctonia solani* AG-1 IA strain YN-7 polygalacturonase 5 (PG5)
NAIMCC-F-03249	MT882064	332	*Rhizoctonia solani* AG-1 IA strain YN-7 polygalacturonase 5 (PG5)
NAIMCC-F-03253	MT882065	315	*Rhizoctonia solani* AG-1 IA strain YN-7 polygalacturonase 5 (PG5)
NAIMCC-F-03254	MT882066	300	*Rhizoctonia solani* AG-1 IA strain YN-7 polygalacturonase 5 (PG5)
NAIMCC-F-03220	MT882067	317	*Rhizoctonia solani* AG-1 IA strain YN-7 polygalacturonase 5 (PG5)
NAIMCC-F-03221	MT882068	308	*Rhizoctonia solani* AG-1 IA strain YN-7 polygalacturonase 5 (PG5)
NAIMCC-F-03222	MT882069	319	*Rhizoctonia solani* AG-1 IA strain YN-7 polygalacturonase 5 (PG5)

Supplementary Table 2. Colony characteristics along with rate of growth and properties of sclerotia.

**Isolates**	**Colony colour**	**Colony growth diameter (cm) at different intervals**			**Formation of sclerotia**	**colour**	**Size**
		**24h**	**48h**	**72h**			
PU RS1	White brown	1.2cm	2.5cm	3.7cm	Peripheral	Dark brown	micro
PU RS2	Light brown	.75cm	2.8cm	5.4cm	Central	Dark brown	micro
PU RS3	White brown	1.5cm	2.5cm	4.2cm	Peripheral	Dark brown	micro
PU RS4	White brown	1.2cm	2.3cm	3.5cm	Peripheral	Dark brown	micro
PU RS5	White brown	0.5cm	2.4cm	3cm	Peripheral	Deep dark brown	micro
PU RS6	White brown	0.85cm	1.8cm	3.4cm	Peripheral	Light brown	micro
MRS 1	White brown	1.3cm	2.8cm	3.2cm	Peripheral	Light brown	micro
MRS 2	White brown	1.5cm	2.0cm	4.0cm	Peripheral	Light brown	micro
MRS 3	White brown	1.25cm	2.0cm	4.5cm	Scattered	Light brown	micro
MRS 4	Light brown	1.5cm	2.4cm	4.2cm	Central	Light brown	micro
MRS 5	Light brown	0.5cm	2.6cm	4.8cm	Peripheral	Light brown	micro
RS-14	Light brown	0.9cm	2.5cm	4.8cm	Central	Dark brown	micro
RS-15	Light brown	1.0cm	2.8cm	4.2cm	Scattered	Dark brown	micro

RS-16	Yellowish brown	1.4cm	1.8cm	4.0cm	Central	Dark brown	micro
RS-17	Light brown	0.5cm	1.8cm	3.5cm	Peripheral	Dark brown	micro
RS-18	Pale brown	0.5cm	1.8cm	3.9cm	Central	Dark brown	micro
RS-19	Yellowish brown	0.5cm	1.8cm	4.0cm	Scattered	Dark brown	micro
RSDSR 1	Yellowish brown	1.2cm	2.8cm	3.5cm	Scattered	Light brown	micro
RSDSR 2	Yellowish brown	1.2cm	3.0cm	4.5cm	Central	Light brown	macro
RSDSR 3	Yellowish brown	1.2cm	3.0cm	4.0cm	Central	Light brown	macro
RSDSR 4	Yellowish brown	1.2cm	2.5cm	3.8cm	Scattered	Light brown	macro
RSDSR 5	Yellowish brown	1.2cm	2.5cm	4.8cm	Scattered	Light brown	macro
RSDSR 6	Yellowish brown	1.2cm	2.6cm	4.7cm	Scattered	Light brown	macro
RSDSR 7	Pale brown	1.2cm	2.9cm	4.0cm	Scattered	Light brown	macro
RSDSR 8	Pale brown	1.5cm	3.0cm	4.5cm	Scattered	Light brown	macro
RSDSR 9	Pale brown	1.3cm	2.1cm	4.5cm	Scattered	Light brown	macro
RSDSR	Pale brown	0.75cm	2.0cm	4.8cm	Central	Light brown	macro

10							
RSDSR 11	Pale brown	0.75cm	1.8cm	3.5cm	Central	Deep dark brown	micro
RSDSR 12	White brown	0.75cm	1.3cm	4.9cm	Central	Deep dark brown	micro
RSDSR 13	White brown	0.75cm	1.3cm	4.9cm	Central	Deep dark brown	micro
RSDVS 1	Light brown	0.75cm	1.5cm	4.0cm	Central	Deep dark brown	micro
RSKVN 1	Light brown	0.5cm	2.5cm	3.9cm	Central	Deep dark brown	micro
RSHVN 1	Light brown	0.5cm	1.2cm	4.0cm	Central	Deep dark brown	micro
RSMIR 1	White brown	0.5cm	2.0cm	5.0cm	Scattered	Deep dark brown	micro
RS-36	White brown	1.2cm	2.5cm	5.2cm	Scattered	Deep dark brown	micro
RS-37	Pale brown	1.2cm	2.0cm	4.8cm	Central	Deep dark brown	micro
RS-38	Light brown	1.0cm	2.0cm	4.0cm	Central	Deep dark brown	micro
RS-39	Light brown	1.0cm	2.0cm	4.0cm	Central	Deep dark brown	micro

RS-40	Light brown	1.0cm	1.8cm	4.5cm	Central	Deep dark brown	macro
RS-41	Pale brown	1.0cm	1.9cm	4.7cm	Scattered	Dark brown	macro
RS-42	Light brown	1.5cm	2.7cm	4.9cm	Scattered	Dark brown	macro
RS-43	Light brown	1.6cm	2.5cm	5.0cm	Scattered	Dark brown	macro
RS-44	Light brown	0.5cm	2.0cm	5.3cm	Scattered	Dark brown	micro
RS-45	Light brown	1.0cm	2.5cm	5.0cm	Scattered	Dark brown	micro
RS-46	White brown	1.0cm	2.0cm	4.0cm	Scattered	Dark brown	micro
RS-47	White brown	1.0cm	2.9cm	4.3cm	Central	Dark brown	micro
RS-48	Light brown	1.0cm	2.5cm	4.0cm	Central	Dark brown	macro
RS-49	Light brown	1.2cm	2.5cm	4.5cm	Central	Dark brown	micro
RS-50	Light brown	1.5cm	2.6cm	4.9cm	Central	Dark brown	micro
RS-51	White brown	1.0cm	2.7cm	5.0cm	Central	Dark brown	micro
AG1L1	White brown	1.0cm	2.4cm	4.9cm	Central	Dark brown	micro

Supplementary Table 3. Raw data output files of qPCR

File 1. Schematic diagram of the q-PCR reaction set-up with letter codes

Letter code	1	2	3	4	5	6	7	8	9	10	11	12
A	RSPG1F/R (DNA conc. -100ng)			RSPG2F/R (DNA conc. -100ng)			RSPG4F/R (DNA conc. -100ng)			RSPG4F/R (DNA conc. -100ng)		
B	RSPG1F/R (DNA conc. -10ng)			RSPG2F/R (DNA conc. -10ng)			RSPG4F/R (DNA conc. -10ng)			RSPG4F/R (DNA conc. -10ng)		
C	RSPG1F/R (DNA conc. -1ng)			RSPG2F/R (DNA conc. -1ng)			RSPG4F/R (DNA conc. -1ng)			RSPG4F/R (DNA conc. -1ng)		
D	×			×			×			×		
E	RSPG1F/R (DNA conc. -0.1ng)			RSPG2F/R (DNA conc. -0.1ng)			RSPG4F/R (DNA conc. -0.1ng)			RSPG4F/R (DNA conc. -0.1ng)		
F	RSPG1F/R (DNA conc. - 0.01ng)			RSPG2F/R (DNA conc. - 0.01ng)			RSPG4F/R (DNA conc. - 0.01ng)			RSPG4F/R (DNA conc. - 0.01ng)		
G	Reference DNA with actin as target gene			NTC			×			×		
H	×			×			×			×		

File 2. Tabular results

Well	Well Type	Dye	Target	Replicate	Tm Product 1 (-R'(T))
A1	Unknown	SYBR	SYBR	---	84
A2	Unknown	SYBR	SYBR	---	84
A3	Unknown	SYBR	SYBR	---	84
A4	Unknown	SYBR	SYBR	---	87
A5	Unknown	SYBR	SYBR	---	87
A6	Unknown	SYBR	SYBR	---	87
A7	Unknown	SYBR	SYBR	---	83.5
A8	Unknown	SYBR	SYBR	---	83.5
A9	Unknown	SYBR	SYBR	---	83.5
A10	Unknown	SYBR	SYBR	---	84.5
A11	Unknown	SYBR	SYBR	---	84.5
A12	Unknown	SYBR	SYBR	---	84.5
B1	Unknown	SYBR	SYBR	---	83.5
B2	Unknown	SYBR	SYBR	---	84
B3	Unknown	SYBR	SYBR	---	84
B4	Unknown	SYBR	SYBR	---	87
B5	Unknown	SYBR	SYBR	---	87
B6	Unknown	SYBR	SYBR	---	81
B7	Unknown	SYBR	SYBR	---	84
B8	Unknown	SYBR	SYBR	---	83.5
B9	Unknown	SYBR	SYBR	---	83.5
B10	Unknown	SYBR	SYBR	---	84.5
B11	Unknown	SYBR	SYBR	---	84
B12	Unknown	SYBR	SYBR	---	84.5
C1	Unknown	SYBR	SYBR	---	84
C2	Unknown	SYBR	SYBR	---	84
C3	Unknown	SYBR	SYBR	---	84.5

C4	Unknown	SYBR	SYBR	---	87
C5	Unknown	SYBR	SYBR	---	87
C6	Unknown	SYBR	SYBR	---	87
C7	Unknown	SYBR	SYBR	---	83.5
C8	Unknown	SYBR	SYBR	---	83.5
C9	Unknown	SYBR	SYBR	---	83.5
C10	Unknown	SYBR	SYBR	---	95
C11	Unknown	SYBR	SYBR	---	84.5
C12	Unknown	SYBR	SYBR	---	84.5
E1	Unknown	SYBR	SYBR	---	84
E2	Unknown	SYBR	SYBR	---	84
E3	Unknown	SYBR	SYBR	---	84
E4	Unknown	SYBR	SYBR	---	87
E5	Unknown	SYBR	SYBR	---	80
E6	Unknown	SYBR	SYBR	---	87
E7	Unknown	SYBR	SYBR	---	83.5
E8	Unknown	SYBR	SYBR	---	83.5
E9	Unknown	SYBR	SYBR	---	83.5
E10	Unknown	SYBR	SYBR	---	84
E11	Unknown	SYBR	SYBR	---	84
E12	Unknown	SYBR	SYBR	---	84.5
F1	Unknown	SYBR	SYBR	---	84
F2	Unknown	SYBR	SYBR	---	84.5
F3	Unknown	SYBR	SYBR	---	84
F4	Unknown	SYBR	SYBR	---	72.5
F5	Unknown	SYBR	SYBR	---	81.5
F6	Unknown	SYBR	SYBR	---	81
F7	Unknown	SYBR	SYBR	---	83
F8	Unknown	SYBR	SYBR	---	83
F9	Unknown	SYBR	SYBR	---	83.5

F10	Unknown	SYBR	SYBR	---	95
F11	Unknown	SYBR	SYBR	---	84
F12	Unknown	SYBR	SYBR	---	84.5
G1	Unknown	SYBR	SYBR	---	85
G2	Unknown	SYBR	SYBR	---	85
G3	Unknown	SYBR	SYBR	---	85
G4	Unknown	SYBR	SYBR	---	85
G5	Unknown	SYBR	SYBR	---	85
G6	Unknown	SYBR	SYBR	---	84
G7	NTC	SYBR	SYBR	---	65
G8	NTC	SYBR	SYBR	---	65
G9	NTC	SYBR	SYBR	---	90
G10	NTC	SYBR	SYBR	---	85.5
G11	NTC	SYBR	SYBR	---	95
G12	NTC	SYBR	SYBR	---	95

File 3: Thermal profile of qPCR

Segment	Plateau	Temperature	Duration	Cycle	Data Marker	Time Step	Temperature Step
Hot Start	1	95	0:03:00	1	No	---	---
Amplification	1	95	0:00:10	40	No	---	---
Amplification	2	52	0:00:30	40	Plateau	---	---
Amplification	3	72	0:00:15	40	No	---	---
Melt	1	95	0:00:30	1	No	---	---
Melt	2	65	0:00:30	1	No	---	---
Melt	3	95	0:00:30	1	Ramp	---	---

File 4: Amplification plots

Cycles	Fluorescence (ΔR)					
	A1	A2	A3	A4	A5	A6
1	-23.55964227	-21.53543443	-23.44480535	-20.60416918	-26.51497373	-11.95067033
2	-11.1559738	-11.25216496	-11.85896803	-16.93995238	-17.71238719	-8.878378981
3	-6.486705324	-6.964095496	-5.73553071	-14.42533557	-12.46740064	-6.822887627
4	-1.23071685	-2.226266028	-1.438973391	-10.67823877	-8.619534089	-5.791556274
5	0.995735623	0.602475439	0.399727928	-7.714565974	-6.962611542	-4.36841692
6	2.133624897	1.139351307	0.981482047	-3.461081973	-4.423301794	-2.671747966
7	1.447894331	1.036036454	1.220275526	-1.706320532	-2.145703406	-0.842011492
8	-0.620272843	-0.453689663	0.539087437	1.206658636	1.45603015	0.669044405
9	-2.129651307	-0.548736176	-0.394689094	1.451536838	2.070248467	0.742977692
10	-2.00375935	-1.242129021	-1.962979627	0.994438393	0.551790771	-0.134249659
11	-2.639055567	-2.376255213	-2.908690648	-0.536675575	-0.590705176	0.310866341
12	-0.699535335	-1.71819734	-2.256788567	-1.822987978	-1.884543976	-0.769781118
13	-0.397289448	-0.789849323	-0.160847964	-1.275143171	-0.733458997	-0.691112597
14	1.49246486	4.350993536	4.543422964	1.68849339	1.276342167	0.714266428
15	0.772265984	11.38839339	13.65816766	10.70211974	10.14687132	8.016850749
16	1.647578154	24.04760361	29.17667318	27.20890241	27.96128932	25.36421823
17	4.520001898	44.01048731	55.85802561	55.82431429	60.93663068	55.4999834
18	11.91095016	75.55846778	94.65269959	99.96008328	111.1329345	103.4023847
19	23.80502565	121.0842023	150.1026073	161.2216495	182.0056154	169.6157929
20	39.70343149	179.922507	217.706226	241.2124467	273.6577643	256.0447296
21	62.90332883	250.8188765	299.0716337	337.3742494	383.4010822	358.1789733
22	91.16439056	329.189373	387.8201413	445.5160995	506.5185275	473.6973843

23	125.4979835	412.4663079	482.5976268	558.8881601	635.1290321	594.23369
24	162.8583155	496.8193559	575.4575278	668.1022723	760.312974	712.8504081
25	201.8385015	577.9689141	662.9397075	768.330837	878.1102152	825.8867877
26	242.7480062	655.092997	744.562826	857.3307025	983.5448037	927.4231821
27	283.7673455	727.3712869	819.1385881	934.8877186	1077.629522	1017.143512
28	324.7945153	793.2753232	886.3330666	1002.710425	1157.771735	1093.200631
29	364.2452182	851.7353501	943.8418171	1057.8977	1226.255473	1159.761896
30	401.1821939	903.0317244	995.3371653	1105.811026	1284.819015	1216.691348
31	436.7011307	951.0065663	1040.492687	1145.142476	1333.866823	1264.195266
32	469.4337142	995.0843713	1080.777564	1180.302761	1375.827445	1304.087714
33	497.9254194	1031.718462	1114.020346	1208.312439	1413.067483	1339.772763
34	523.8116782	1064.884403	1144.28058	1234.057761	1445.745967	1371.894037
35	547.9286721	1093.267971	1169.735886	1254.120091	1474.368023	1398.461076
36	571.5707237	1122.801435	1194.433696	1272.392952	1496.266483	1420.604514
37	591.9639339	1146.808403	1215.019022	1287.17132	1517.208938	1440.352384
38	611.0123874	1170.740051	1235.030351	1300.692896	1533.215473	1456.73642
39	629.1161701	1192.032322	1253.274216	1313.306646	1549.963344	1473.08892
40	648.581941	1215.602409	1271.299649	1324.329478	1562.483681	1485.584179
**Cycles**	**Fluorescence (ΔR)**					
	A7	A8	A9	A10	A11	A12
1	-2.327641931	3.31788764	14.75503956	-10.25668638	-7.218996153	-6.440446964
2	0.396627169	6.936323193	16.55346691	-4.457949756	-1.977621964	-3.823590042
3	1.473696268	6.571558746	14.62549425	-1.250413134	-1.049847775	-1.632333121
4	0.658125367	6.130954299	13.0338416	1.311683489	1.193606415	0.5802038
5	0.934586467	5.878541852	10.56417294	2.626452111	2.037476604	1.111876721
6	-0.949074034	4.648599805	7.186165087	2.362667133	1.864565993	1.311632842
7	-0.646352454	2.660790238	4.850886193	1.533705835	0.408382422	0.508832803
8	-1.738378779	0.325901247	0.74248525	-1.724001446	-1.907811901	-0.160861827
9	-1.672078269	-1.229977132	-	-3.780493189	-2.452662966	-0.404446609

			1.711994311			
10	-1.653061972	-1.299469275	-3.190314004	-4.682491021	-2.61524753	-0.936188338
11	0.388162849	-1.715882047	-3.942617448	-4.913346962	-2.547110142	-2.040339488
12	-0.175627467	-1.704401699	-2.354485668	-2.179075743	0.243624936	-0.716603911
13	0.744020893	-0.676726852	-0.553063594	0.682449285	4.825023943	2.378227127
14	2.239353961	3.639765519	6.159103584	8.762450508	14.10907527	9.912854603
15	10.94651171	13.61726029	18.09607784	21.51159773	29.29178985	23.08966046
16	28.41117133	34.58471906	43.66016254	47.63826939	57.59476755	46.48286128
17	60.86969626	71.28237109	86.99463073	90.37427515	103.5015305	86.44777677
18	110.8784946	128.1240546	152.2085977	153.9076526	169.7531032	143.9703142
19	180.7961208	204.940583	239.9525412	232.1223712	250.7943948	215.5187189
20	272.6055671	305.7808867	351.1783797	325.4328324	347.4625922	297.9838215
21	377.1751431	421.2209145	480.6065924	435.2987103	461.4141145	393.4374371
22	491.875109	545.1856421	617.1716591	557.2948201	587.9476829	499.2720949
23	602.9531788	663.1752545	746.0045714	677.6280597	714.8543256	605.3806637
24	710.7329474	772.2747839	861.7184236	784.6547715	827.5519923	699.6202232
25	809.9286766	869.8012736	962.2620329	874.2876037	922.2824787	776.140763
26	897.1479376	952.2351387	1047.547782	948.9803545	1000.377734	837.943697
27	973.6550971	1023.335871	1118.34791	1008.206313	1064.952507	887.459306
28	1038.224543	1080.951452	1175.799341	1055.550897	1118.096137	927.9739287
29	1096.064025	1131.803376	1225.68391	1092.825847	1160.049442	960.2308892
30	1144.569972	1172.605538	1265.785366	1122.310596	1193.078453	986.23612
31	1184.86322	1209.045017	1300.616828	1148.023378	1221.484541	1007.539472
32	1217.44722	1238.002006	1329.437668	1168.823726	1244.981186	1025.052103
33	1247.646831	1264.789961	1355.002384	1185.487194	1262.571357	1036.866214
34	1273.827715	1286.047611	1373.913752	1197.340799	1276.798344	1047.582476
35	1297.527975	1306.165394	1389.843225	1208.605056	1288.9714	1054.739465
36	1316.128365	1322.149142	1402.64565	1220.18947	1301.461034	1061.76503

37	1333.612657	1336.478109	1414.226286	1228.120046	1309.003196	1065.052454
38	1348.053755	1348.649313	1423.737154	1234.983886	1316.61918	1068.965966
39	1362.177933	1360.627955	1433.016125	1239.52499	1322.377961	1072.135263
40	1374.1278	1373.392086	1443.257352	1246.697491	1330.658919	1076.675768
**Cycles**	**Fluorescence (ΔR)**					
	B1	B2	B3	B4	B5	B6
1	-112.7468664	-23.32394488	-32.77642605	-59.84621274	-52.96118246	-24.44155122
2	-59.69454483	-12.70985957	-19.03623983	-35.18678553	-20.82633434	-12.66323972
3	-37.0614233	-7.506974258	-10.1432536	-22.33615832	-11.66828622	-6.521728209
4	-18.30734177	-2.333528946	-3.682907372	-12.82009111	-4.686398098	-2.948376701
5	-9.412908235	-0.048211635	-1.078529145	-6.932695895	-1.735101976	-1.216017194
6	-3.466212304	0.853592076	1.268127482	-2.437947083	-0.225156254	-0.165488086
7	-0.680993493	1.101067468	1.59204619	-0.147461951	1.190400988	0.780476542
8	1.082382374	0.340974475	2.459872993	2.223641165	2.498810454	1.969162193
9	1.609094229	0.048502142	1.431933832	2.370015142	3.166912669	2.185479153
10	2.284104691	-0.551959736	-0.466376903	1.687566887	1.885523879	1.45586658
11	0.541442073	-1.720495392	-2.917915146	0.194408359	0.086175437	0.742594362
12	0.344904561	-1.064243712	-4.054173205	-1.22665667	-3.206663136	-1.356595691
13	-0.225942546	-1.55664932	-3.438020729	-2.595445055	-4.701791666	-2.129701241
14	1.237573448	-0.113828918	-2.408330109	-4.33935941	-5.936076214	-3.514773457
15	-0.166899856	0.086305227	-1.145449754	-4.347843631	-4.358649956	-3.757022106
16	-1.138098399	2.624947323	0.846776178	-3.484267019	-2.56671274	-4.293099374
17	-1.39623985	8.182753759	6.831509172	0.900807786	2.430468828	-2.659377653
18	-0.02511493	20.01209465	19.36061269	8.764594397	9.511601457	1.149538142
19	3.614474682	42.8995753	43.19709173	27.42842301	26.45057317	9.42745255
20	11.23361051	80.6529908	82.60391998	59.54800238	58.17790291	23.23420549
21	29.30234851	138.1912212	141.5862932	117.1187403	114.4344721	46.24052587
22	56.8569162	210.0596016	218.1978452	199.8708601	198.1545607	86.19252744
23	96.59857025	297.3509749	313.250342	311.549488	309.8732012	147.7735787

24	144.6748075	399.1929769	425.5168635	445.4496938	446.2842559	231.4695762
25	200.8553787	510.4297031	547.3143789	595.7795169	601.0335038	333.5143729
26	260.5237333	622.8554999	670.260898	753.639579	763.5888731	447.2519186
27	322.3105114	723.5980557	780.7434166	905.4916124	919.9731055	569.397774
28	383.0185308	811.4417774	878.5629359	1043.448088	1061.284335	692.7638411
29	439.9344833	883.1690841	958.1570552	1160.623846	1182.346736	812.0556125
30	491.8762706	942.093341	1025.973495	1259.464349	1284.744771	922.3765671
31	537.4648114	990.6337049	1080.989318	1340.881657	1369.360167	1019.888499
32	579.18787	1030.93668	1128.689483	1408.947276	1439.486162	1105.844463
33	614.4671828	1063.315399	1166.166392	1462.857918	1497.557749	1182.92743
34	645.28465	1090.461789	1198.135518	1508.867226	1545.920574	1249.724603
35	671.7209989	1113.176862	1223.158437	1544.465273	1585.130766	1308.090017
36	696.588755	1133.959206	1246.290558	1575.4008	1617.168678	1356.311922
37	717.666569	1149.668741	1264.855278	1600.121571	1644.541608	1401.218773
38	736.8726759	1163.777168	1282.128357	1622.266887	1666.147085	1439.433911
39	754.111067	1175.85585	1297.282729	1642.59772	1686.771649	1477.383984
40	772.5528863	1189.013267	1312.735671	1661.22159	1702.91092	1510.911371
**Cycles**	**Fluorescence (ΔR)**					
	B7	B8	B9	B10	B11	B12
1	-14.77851397	-25.26270786	-20.07743618	-16.57034831	-11.0043801	-9.432478845
2	-2.691709103	-16.4494573	-8.988519287	-5.816144359	-4.993832475	-4.025236497
3	-0.514504236	-12.91220675	-4.670002393	-1.041940405	-1.392884848	-0.829194149
4	1.28318063	-7.78615619	-0.787965499	2.256263548	1.628542778	1.477408199
5	1.823041496	-3.597545633	1.452695395	3.463267501	2.452146404	2.563882547
6	1.435433563	-1.078663076	1.677785088	3.556831454	2.720281231	2.628443295
7	1.410767069	0.518785881	1.371485342	2.209467407	1.637757497	1.844595723
8	0.673195104	0.198002518	-0.644206532	-1.04877024	-0.225991709	0.686683768
9	0.265630332	0.309285971	-1.508054719	-2.977368206	-2.116117721	0.484277271
10	-0.418514096	0.723336323	-1.283412595	-4.022212957	-2.967764189	-1.001841011

11	-0.891973016	1.084353418	-1.010703672	-4.624379129	-4.416990109	-3.153600558
12	-3.378610765	0.095317242	-0.710683326	-4.161258942	-3.178036011	-4.895230715
13	-4.82574718	-0.77433624	-0.995587336	-3.596545767	-2.920961799	-5.687963246
14	-5.867619092	-2.090123838	-0.592013932	-1.998456722	-1.122627562	-4.208890375
15	-4.020537838	-1.271261719	-0.167729917	-1.773373906	-0.812417296	-2.285676899
16	0.68538695	2.285303519	3.198391703	1.783782838	1.908420181	2.18072578
17	12.34087108	12.81633932	11.75702297	10.93275312	7.413758305	10.84459442
18	35.95803578	36.08980987	31.74252368	32.4665009	20.958122	28.85659409
19	80.95744847	81.30666147	69.05190021	73.59556688	45.46719094	63.17771316
20	160.2256469	162.0606762	135.9114258	147.520652	90.17700604	123.8302823
21	277.824052	279.3107998	233.3457564	265.9240046	162.5199114	224.2109654
22	425.5422556	424.9675779	355.8050777	420.5822145	257.2295841	359.4035612
23	589.3504603	583.8049085	490.3843582	591.3870494	362.9392283	513.3041624
24	763.8006249	751.7596806	633.9926288	763.4721807	468.3222202	666.9087472
25	940.5971817	920.7740515	779.2306891	932.8426963	574.039876	815.7957298
26	1104.991409	1077.02383	913.4005054	1097.126348	677.7591342	959.2799887
27	1243.174449	1208.732611	1027.882631	1244.449704	772.9456455	1089.140182
28	1352.834785	1312.780273	1118.613569	1365.763628	851.825928	1197.395018
29	1440.948343	1396.987512	1194.656732	1462.483606	914.1384153	1281.00397
30	1509.248005	1461.294442	1252.012103	1535.482909	961.0760979	1345.962673
31	1563.275532	1512.253226	1300.49236	1593.219288	998.0252604	1395.662149
32	1604.285852	1550.56232	1337.060035	1636.358946	1027.301758	1434.97973
33	1639.238305	1583.471846	1369.070172	1670.126676	1049.246019	1462.769087
34	1666.175742	1608.37152	1393.586284	1694.300676	1066.389299	1483.57642
35	1688.298603	1630.98931	1414.892084	1714.281544	1078.305936	1498.081703
36	1705.255546	1647.948753	1433.657017	1730.705041	1089.017048	1509.530172
37	1721.016389	1663.92015	1449.471714	1742.978436	1096.241726	1517.106868
38	1733.70483	1675.362269	1463.58819	1753.110336	1103.328013	1524.697846
39	1745.731376	1686.588338	1476.02136	1761.584174	1109.611495	1531.673064
40	1755.884644	1696.721954	1489.211493	1772.3936	1116.805946	1540.752536

**Cycles**	**Fluorescence (ΔR)**					
	C1	C2	C3	C4	C5	C6
1	9.107710784	-15.37477782	-25.85439938	-24.32394285	-15.52508708	-17.08388897
2	11.65668197	-7.403562737	-13.24208796	-12.82971504	-6.185547768	-9.696030998
3	11.17045316	-2.637947654	-7.831376538	-7.119487223	-1.441208457	-5.997773029
4	11.18598434	1.31294743	-1.910585118	-3.370059408	0.332890855	-2.71503506
5	8.694827528	2.859778513	-0.328097698	-1.369591594	1.993902166	-1.053321091
6	6.602685114	2.162852797	1.888744922	-0.11107578	2.838247878	-0.198915923
7	3.689007981	0.736362921	1.764797782	1.049257635	3.296642869	0.369822686
8	0.490826783	-0.084791148	2.09956373	2.641564169	3.714514597	1.919966223
9	-1.528562172	-0.130790887	1.457914343	3.300628848	2.447091527	3.209257434
10	-2.169093491	-0.500692598	-0.287275493	1.459439779	0.834504846	3.042659164
11	-2.498094834	-2.580343838	-1.648456485	-1.068448411	-0.584173518	2.932712534
12	-0.889018654	-2.866725378	-1.953543799	-3.433727174	-3.23310294	0.742918335
13	-0.430020975	-2.936402884	-2.370610608	-3.503823876	-4.48930091	-1.051515049
14	1.586576508	-2.404085643	-2.99885458	-3.48236228	-5.913002802	-3.582845785
15	0.684678252	-2.908028645	-3.491729885	-2.583552613	-4.691659187	-3.982483827
16	1.110600809	-2.498824748	-2.999766888	-2.490961671	-3.574807256	-3.703162802
17	0.018388381	-1.514243518	-1.683762498	-0.184144861	-0.74984456	-2.261711424
18	-0.111886881	2.568042557	1.594017832	1.404273378	2.395841953	0.147957838
19	0.046598293	8.784945068	8.628961628	6.891857076	10.54729538	6.183696747
20	1.855222986	21.04831184	21.4076933	15.61559415	26.16404696	19.33829316
21	9.451627672	45.54789474	47.88661525	36.966395	55.07501154	41.84187501
22	24.13561626	89.30401373	96.45433283	75.48983927	108.3378784	83.23902544
23	49.19467763	157.3586832	176.5798476	142.173225	197.1299682	152.8846867
24	84.14627033	243.5243699	282.0346809	235.7231277	322.0982832	252.3587638
25	130.3513839	341.7719702	402.2669374	350.878322	469.6076877	377.2482262
26	190.5857078	455.0581566	535.1205422	481.7278781	628.6605552	514.7864489
27	261.676578	580.768443	681.4539013	625.6373649	795.7303332	668.9375008

28	337.9445996	713.3712666	835.6074809	777.0977102	967.7121859	827.5408704
29	410.6193606	834.2374177	979.4210555	921.6802133	1130.479836	983.9572695
30	475.2108999	938.9347417	1103.930673	1049.59732	1273.18706	1119.626738
31	531.8671426	1025.050966	1205.111499	1155.605778	1389.639358	1233.909426
32	580.5196817	1096.017204	1289.165774	1240.899428	1482.628586	1326.765373
33	621.3844208	1151.037226	1353.328981	1308.668386	1556.274215	1403.658614
34	654.6908592	1196.638007	1406.688664	1362.989445	1614.35851	1465.073967
35	681.5280774	1232.965697	1447.909429	1404.915985	1660.061818	1513.4012
36	705.1597914	1263.75492	1482.941706	1438.074041	1694.976663	1551.415232
37	723.6565606	1286.181831	1509.508501	1464.399497	1724.057617	1584.149
38	740.8852399	1305.828587	1531.744502	1486.804736	1746.8141	1609.764075
39	756.0203236	1323.434363	1551.674079	1506.968288	1768.848582	1633.671158
40	773.2746208	1342.253545	1571.552626	1524.348086	1786.488987	1652.489097
**Cycles**	**Fluorescence (ΔR)**					
	C7	C8	C9	C10	C11	C12
1	-16.4502927	-20.82447219	-23.14353569	-30.95195461	-17.15691692	-8.212244724
2	-6.318378668	-8.813849103	-8.098465743	-18.33948639	-8.641851868	-1.80168475
3	-1.983264638	-5.646426015	-2.070195792	-11.31661817	-3.043586817	1.742475225
4	-0.508310608	-2.786842928	2.33391416	-4.989269955	1.050518234	4.854955199
5	0.235251422	-0.757467841	2.809832111	-1.518945738	2.460431285	5.907819174
6	0.460503052	-0.155702353	1.975279663	0.640869679	2.432673937	5.862423949
7	1.635814202	0.994499614	0.692994734	0.367178535	1.180544108	3.985453683
8	1.697475177	0.568866957	-1.54093077	-1.05531568	-0.403994297	2.32251657
9	1.726418019	0.737754672	-3.054730886	-1.794271821	-0.899888913	0.536071079
10	1.526087201	1.710379536	-2.214834039	-0.226280962	-1.244536486	-1.432269457
11	2.273358024	2.562655904	-1.360172722	0.739806911	-2.141205894	-3.861690678
12	0.164294443	2.151610003	-0.431819119	2.846903588	-2.518030259	-5.419707044
13	-1.126515691	1.063829949	-0.485774164	2.461821428	-2.701289983	-6.695658577
14	-3.424942016	-0.81196139	-0.721452481	3.006505262	-1.54186777	-6.940916172

15	-3.961240889	-2.180701816	-1.789937183	1.438706527	-1.875196241	-7.323622013
16	-4.546637511	-2.805634317	-3.061327816	1.034364477	-1.438538462	-6.527678715
17	-2.989428192	-2.98039505	-2.339860912	-0.15978275	-1.34643357	-5.323495239
18	0.786482765	-1.316359844	0.539596314	-0.279199676	1.830757995	-1.201933899
19	8.034655239	4.005468903	8.64922311	-1.341631576	7.156056317	5.784751048
20	22.22102034	17.62661555	25.63648189	-1.577720411	18.92799333	20.32793629
21	46.88947628	42.03918448	59.04530097	-1.237143628	44.21887942	51.75544631
22	92.8419889	85.06990144	119.1139585	-0.815965108	91.10288307	109.8711214
23	168.547731	155.5825325	215.9988958	-0.663333117	169.2093001	211.5012943
24	279.6609304	258.5151759	348.3790567	-0.548290085	283.0788232	361.7719999
25	418.6062671	389.2282047	503.4215184	-0.89447415	420.9454503	548.0737116
26	576.1995228	538.981313	666.4954848	-0.740421427	566.3641193	744.3097311
27	741.2887896	695.6985142	833.3082124	-1.378566765	703.8926176	931.7388133
28	902.0068426	848.01655	992.2749932	-1.075104358	832.5534901	1106.19337
29	1045.949855	986.2475713	1137.020337	-0.541760014	949.2628033	1262.691633
30	1165.863616	1104.581357	1253.352204	-0.574117733	1049.008279	1396.803733
31	1263.41652	1203.918292	1348.957088	0.126360522	1130.370676	1504.647342
32	1341.491401	1285.87641	1422.93388	0.660265559	1194.06369	1589.36002
33	1406.798506	1355.559394	1484.039657	1.307423148	1242.546211	1652.192813
34	1460.056452	1413.911588	1531.445613	-0.656083397	1278.252757	1700.023443
35	1504.551011	1463.742598	1571.737402	-1.119072258	1305.36201	1736.077664
36	1540.68306	1504.203213	1604.866392	-1.404090409	1327.196608	1765.37617
37	1572.38993	1538.885511	1632.13999	0.046589119	1342.656818	1785.968251
38	1598.139262	1567.938068	1654.80995	0.480002324	1355.787218	1803.467905
39	1622.733319	1596.565858	1675.512403	1.410933009	1366.69004	1817.736954
40	1643.788514	1624.235006	1697.46253	3.033840975	1379.857513	1833.41461
**Cycles**	**Fluorescence (ΔR)**					
	E1	E2	E3	E4	E5	E6
1	-16.30203668	-36.0828097	-76.31828382	-45.67936633	-122.6748864	-21.40151219

2	-8.575705506	-28.49361623	-50.02866167	-30.12561266	-84.3082404	-10.72782113
3	-4.705374334	-24.22122277	-33.73743953	-19.666259	-62.68559445	-5.962930071
4	-0.842243163	-18.9329893	-19.69629738	-12.35218534	-45.77574849	-3.45259901
5	0.848248008	-14.70494784	-10.90485123	-7.08604768	-33.75726253	-1.37493995
6	1.90277118	-8.88577677	-4.413360287	-2.858553218	-23.85960858	-0.23472729
7	0.795572751	-4.560418185	-1.431799581	-0.448374597	-14.76439302	1.631461691
8	-1.071163598	-1.415596176	1.587784037	2.590612216	-5.653831545	3.055356655
9	-2.32215185	0.794356021	2.712986189	2.5918975	0.499311436	3.53598808
10	-1.201898068	2.581226942	2.666916631	2.311404117	3.064039901	1.939507994
11	-1.484246259	2.096507645	1.007716438	-0.232985306	4.719601764	2.338952898
12	0.827133562	2.672854048	0.391635775	-2.086509671	3.675647403	-0.477839502
13	0.976738591	1.607095548	0.261552891	-3.054640232	3.709956478	-1.138694365
14	2.212734264	2.425129208	0.637293469	-2.90751902	2.620014994	-4.411609182
15	0.833653107	0.691500319	0.411398296	-1.360268693	3.320876086	-4.575748481
16	0.148834713	-1.175702648	-0.633659335	-1.109942188	2.755047582	-5.440544668
17	-0.320146495	-2.97995294	-1.362876609	-0.238974625	3.694041673	-4.623717129
18	-0.407107712	-3.198327512	-1.592758302	-0.103263614	3.480662365	-4.610696223
19	-0.774497494	-2.686936406	-1.659604808	0.209524297	3.937772895	-3.222111898
20	-1.521568991	-1.912417021	-2.19397716	0.010676279	2.698506713	0.080823785
21	1.399337456	5.060681157	3.610752356	0.344916454	-0.545936843	3.841742423
22	7.177903148	20.72612075	18.18979708	3.483447082	-3.330691217	12.07712792
23	18.26159628	52.76974438	51.79152521	14.48945344	-5.424543229	27.49900339
24	36.57784675	107.6274731	109.5526531	36.42981303	-5.288276933	58.45307022
25	66.00163417	190.5236597	201.7501976	75.73053842	-3.167806502	114.5508733
26	108.4934404	299.9903588	324.4669053	136.4902076	2.995528418	199.3838619
27	163.2203579	425.978852	466.574729	218.2137387	16.36427706	313.1926349
28	231.4079013	569.7858066	622.8646086	320.021831	39.58268135	450.5436019
29	313.7345922	726.0608122	790.2691226	433.8396079	76.21209951	608.9981645
30	403.5812378	888.9931203	963.9329746	558.6762339	130.2936516	779.781885
31	492.3597038	1040.750499	1125.071621	680.3185667	198.3478332	948.6521563

32	571.2285248	1173.004353	1264.157094	794.9258106	278.8869675	1103.605569
33	637.5017809	1279.722515	1375.926891	891.8871782	369.9176183	1241.392921
34	692.273995	1366.032834	1467.622919	976.9123527	468.5435628	1359.163688
35	738.2268878	1434.554447	1539.241057	1043.421113	567.3868695	1456.897927
36	777.5157079	1492.436749	1597.828863	1101.039352	662.7927073	1536.021543
37	808.9078491	1538.233448	1642.395025	1145.776205	754.5545238	1603.415728
38	835.5878401	1577.285165	1680.150792	1185.358675	840.3000422	1658.141903
39	859.5578689	1612.500635	1714.17666	1220.685148	923.6924812	1710.148554
40	885.0356955	1647.654645	1747.97272	1254.368228	1001.637515	1754.924616
**Cycles**	**Fluorescence (ΔR)**					
	E7	E8	E9	E10	E11	E12
1	-14.59153324	-25.77634435	-47.78577258	-9.824442911	-9.554733214	-20.0494633
2	-4.751525563	-12.6574616	-28.54354983	-4.721232338	-5.964433036	-12.42739601
3	-1.547517886	-6.908178846	-18.05972709	-3.613221765	-2.723732858	-6.434128713
4	-0.586710209	-3.290416094	-9.537984342	-0.855451193	1.109447319	-0.659421419
5	0.798257468	-0.972877342	-4.160337597	0.43323138	2.191203497	1.345813876
6	0.619417145	0.05831261	-0.803926051	1.958048353	3.441170874	2.35344277
7	1.812647222	1.172188002	0.719419255	2.236274606	1.774495692	2.407655984
8	1.167529779	0.645330722	0.071904272	-0.487590406	-0.441865762	2.471664782
9	2.029156912	-0.193136004	-0.376396016	-1.661191814	-2.551492983	2.346949562
10	1.624463457	0.977928846	2.180974577	-1.725158754	-2.549710611	0.786448654
11	3.267854798	2.688578122	3.179322286	-1.25714608	-2.904299475	-2.498954196
12	1.267598982	4.309050598	5.066999593	0.33918941	-1.507880666	-3.61649457
13	0.148230693	3.8194046	3.020738244	0.267585387	-0.632534571	-4.245442839
14	-3.323689522	1.489699546	1.965555083	1.488058024	0.888798516	-2.743100191
15	-4.289942617	-1.030041013	-1.678200172	0.23335809	0.63511446	-1.716780938
16	-5.425572672	-3.555800484	-2.841454208	-0.257961026	-0.244375628	-1.559408182
17	-4.893944696	-5.120770839	-5.026322418	-1.891638492	-1.20403035	-1.271029403
18	-4.862740506	-5.494787153	-4.71941322	-1.801111465	-0.904879205	-1.33023872

19	-2.598169466	-4.438454835	-4.318471376	-2.594215209	-0.569999814	-0.432764451
20	3.212990186	0.542138017	-0.00036752	-0.419204209	0.4237864	0.786528917
21	11.58014093	8.393652702	7.719975268	5.572703586	4.156499627	8.26152282
22	28.56780762	25.60420386	23.50419824	18.12161369	12.76877962	25.65202065
23	57.99077571	55.06074668	51.18164504	38.74530361	30.10475837	61.27675937
24	114.8249073	110.6382951	105.0505126	75.89534996	61.36139023	121.5314471
25	209.0283318	200.289243	193.5363092	135.9656236	110.3468925	219.9589727
26	335.9878476	320.979072	315.3837756	220.7252139	179.6622994	369.1470558
27	483.3724403	461.691357	459.6269619	318.206713	259.7894613	548.1218179
28	639.3932666	615.4159094	619.0216261	420.3704573	349.9449552	738.0060273
29	801.026355	777.5474066	787.1257298	526.2150323	447.6684665	915.029462
30	954.9091426	933.5627461	948.8020171	633.3322225	551.9112476	1086.862631
31	1088.564322	1070.436243	1093.334629	737.8401019	653.7714862	1244.885592
32	1194.82392	1182.45814	1213.552942	827.4806421	743.6590702	1382.108459
33	1284.358881	1277.881349	1316.479661	902.4258524	817.4756149	1488.809265
34	1353.869797	1354.414499	1399.685201	962.4585288	875.6834207	1572.24564
35	1412.030977	1419.249901	1472.088187	1012.969633	922.5552709	1635.324716
36	1455.117401	1470.967741	1532.786425	1055.993915	962.6381822	1685.879447
37	1495.718926	1516.322519	1584.783204	1089.016518	994.6961146	1722.257848
38	1526.00852	1552.181172	1627.098741	1116.941422	1021.991263	1751.496115
39	1556.589731	1586.47488	1665.998042	1140.329512	1045.957656	1777.106201
40	1580.374885	1617.11218	1703.390802	1166.023904	1071.050176	1802.439191

**Cycles**	**Fluorescence (ΔR)**					
	F1	F2	F3	F4	F5	F6
1	-7.901990333	-21.03583059	-33.07046613	-101.9432769	-32.45638677	-20.36500702
2	-2.080452694	-11.61659399	-19.18864346	-67.20726645	-20.6007555	-8.329950525
3	-1.240515055	-7.682157385	-12.0884208	-51.92725602	-11.79152423	-4.437294026
4	1.073502585	-2.973480781	-6.374118131	-37.5744456	-7.029972961	-2.687517528
5	1.886016224	-0.406916178	-3.493319465	-27.89827517	-3.487237691	-1.99479703

6	1.693045064	1.286074025	-1.456405599	-18.94287275	-1.197801621	-0.742063731
7	0.598676143	0.575926948	-0.154969493	-12.06695192	0.997211408	1.211260927
8	-1.47706929	-0.789562465	1.230594101	-6.351081017	2.72267999	3.416706417
9	-2.529369577	-0.966747801	2.285887641	-3.083116416	3.735355074	3.812694346
10	-1.973256137	-0.437340789	1.291952668	-1.93674306	2.511562569	2.297214929
11	-2.290770923	-0.328954491	-0.977882019	-1.304269214	1.497917846	2.03755053
12	0.538671392	1.236546132	-1.712742352	-1.598893518	-0.920990837	-0.353244334
13	1.222779481	1.409265478	-1.395787756	-0.381717355	-2.178922757	-0.519102288
14	3.307212145	2.794851434	0.438524696	1.152399272	-3.685712116	-3.066198952
15	1.44264288	1.544454457	0.986671706	4.652264085	-2.81007761	-2.244555977
16	0.668338143	0.809434214	1.181057198	6.408666627	-2.107729818	-2.625412816
17	-1.277714074	-0.749707268	0.847457298	8.209526354	-1.163554597	-1.77302169
18	-0.840062881	-1.570597651	0.737508016	7.470585062	-1.605671233	-3.314480933
19	-1.560020502	-2.808662062	-0.033308221	6.832575004	-1.476680754	-3.488060658
20	-2.234759204	-4.982511059	-2.491567725	5.506790989	-1.510727224	-2.466834552
21	-2.731975885	-4.626951779	-3.419489273	4.86363843	-2.86315966	-2.533071377
22	-2.384644378	-2.95266776	-3.778831884	4.21945737	-2.91187668	-2.977839622
23	-0.832898829	2.091242862	0.281608884	3.711596901	-1.363527809	-3.715806737
24	1.928639166	8.872823752	6.139722115	2.23079485	2.964977323	-1.98811991
25	5.773018458	22.27586402	16.64132651	-0.817331399	10.16892689	4.174057932
26	13.6759245	50.55073021	38.23116364	-2.373510804	24.30399659	14.11626476
27	27.80710415	100.5242535	76.76734555	-3.54476568	49.6003792	32.3013756
28	50.99564387	183.4118733	142.710443	-4.140646281	92.11524843	61.69107303
29	83.84131033	296.0220439	230.1241924	-8.144467121	153.7060776	111.9702686
30	121.4298741	432.5595439	338.5134554	-9.514801155	238.155796	188.0682811
31	166.2984428	583.82702	459.9919515	-7.040025875	339.3924843	289.9079566
32	216.3715919	750.4259572	600.2833969	1.53046863	459.3583444	412.2597281
33	270.741658	923.6371818	749.1552788	17.28912884	589.8274328	551.0622514
34	322.4120236	1092.237156	900.1058378	43.30456604	724.3430013	697.2573442
35	368.8018325	1239.237338	1032.78822	78.80899178	847.1685114	841.2211014

36	407.39559	1362.995311	1147.832701	123.8625706	954.0653059	975.8171053
37	438.3740259	1461.384884	1239.295968	173.7237778	1045.838346	1097.493291
38	464.0701872	1542.052335	1316.115411	225.8563412	1122.400891	1203.711963
39	488.2415631	1615.913262	1385.502565	277.2056337	1195.006586	1303.892742
40	513.7446287	1686.868583	1451.623227	326.729871	1260.37057	1396.150379
**Cycles**	**Fluorescence (ΔR)**					
	F7	F8	F9	F10	F11	F12
1	-21.51209158	-26.97726735	-27.61133688	-20.07197148	-18.31264464	-15.48457276
2	-11.2577419	-13.06288944	-13.14459998	-9.990767782	-9.300008237	-8.783817948
3	-7.482592226	-8.158111542	-7.912263072	-6.907964089	-5.20977183	-5.147063133
4	-5.058482549	-3.75285364	-3.293206166	-2.775240395	-0.398415424	-1.007108318
5	-2.600420873	-0.649419738	-1.04368526	-0.432212701	0.972684983	0.020686497
6	-1.405776796	0.893745364	0.609272445	0.362859792	1.599958189	1.926689312
7	0.74297416	2.264491906	1.069010311	-0.109597954	0.390414756	1.785901728
8	1.629862973	1.088700976	-0.029208431	-1.945152788	-0.535257446	2.811397663
9	1.255200732	0.76911884	0.322337537	-1.906833088	-0.771518729	2.860792222
10	0.975855853	1.511470968	1.652245125	0.033642101	-0.813123582	1.54822321
11	2.263264235	2.837451708	3.06777798	0.529323295	-1.677914965	-0.831958792
12	2.083086741	3.692545022	3.896108212	2.316476788	0.731587632	-2.098056105
13	0.722742725	2.570186575	3.224122973	2.072965942	1.431311718	-2.554859079
14	-2.367151771	1.158160289	3.134634115	3.481616687	3.843938899	-1.226986248
15	-2.838989668	-1.507289916	0.661581439	2.214566883	2.057190996	-0.980319387
16	-2.733126341	-2.681358473	-1.171684725	1.542809288	0.573148696	-0.59295856
17	-2.388111449	-4.007835484	-3.553706349	0.254970024	-1.290227501	-1.193700133
18	-2.679725999	-4.466517857	-4.917521763	0.162972869	-2.368929356	-1.363923393
19	-2.850836124	-5.182122992	-5.387447028	-0.613072199	-3.366563123	-2.045663471
20	-0.725171254	-3.975553753	-4.67495302	-1.286758428	-6.191048403	-4.343603249
21	-1.116050499	-2.83593419	-2.747197128	-0.076782471	-5.964690369	-5.267086331
22	-0.350883568	-0.125269689	-1.339905004	0.730397691	-3.293533974	-5.038918014

23	-0.857816225	2.086213865	-0.793653258	1.433751131	2.676750756	-1.305528078
24	1.612040436	10.31207022	2.076297659	0.835780068	11.99582082	4.559236461
25	7.622835043	26.44096494	9.238780336	-0.043221241	26.64447363	13.35132025
26	17.5371751	56.3533419	22.3245092	-0.438693499	58.22880005	33.3551428
27	35.74041184	107.603023	46.45339365	-1.593665996	115.4661777	71.38677696
28	65.58213701	189.8768613	88.57555845	-1.603832731	210.4213004	136.8663212
29	115.8093392	306.2229919	162.7050104	-1.536938361	336.6505823	229.2410098
30	183.8413345	443.1884125	266.034576	-1.825670618	483.2782451	342.8843092
31	269.3113838	595.4921495	395.6056216	-1.570115979	643.4404159	472.9603597
32	362.8300025	748.3874077	538.464986	-1.076829286	819.3891644	618.7766826
33	462.8459459	898.8686335	689.6303101	-0.227138804	1000.20213	772.0276101
34	557.1710681	1029.185357	842.51449	-1.258621153	1171.745255	923.7135131
35	644.2574911	1142.586374	992.4036329	-0.59505731	1318.207255	1055.773332
36	719.5580099	1237.171348	1133.83754	0.231281205	1439.599062	1167.594929
37	788.8536081	1318.209973	1260.581392	0.429183893	1535.160605	1254.843666
38	847.5910413	1384.97612	1372.496186	0.73395435	1611.542057	1325.530185
39	905.1425462	1447.360338	1478.391337	0.391828955	1681.306803	1389.221583
40	956.743939	1505.927861	1581.583319	3.157054033	1748.103666	1449.855556
**Cycles**	**Fluorescence (ΔR)**					
	G1	G2	G3	G4	G5	G6
1	-111.0963394	-95.07148824	-117.330891	-147.8028952	-164.2212688	-161.069092
2	-68.21458558	-51.43609631	-65.53932857	-101.5943189	-118.5330293	-116.2038632
3	-49.14883177	-33.80150439	-45.93096613	-76.18654261	-92.29918973	-94.61943434
4	-30.24227796	-16.39187246	-25.83844369	-50.06372631	-67.34263018	-69.69596553
5	-17.93076415	-7.62739254	-15.08572925	-30.95806201	-46.13240662	-47.76084871
6	-8.770098344	-2.436935016	-7.904144403	-16.11282011	-26.52690627	-25.9555943
7	-3.758610136	-0.490312372	-4.70474708	-7.92309309	-12.39161776	-12.37398276
8	-0.807127047	-0.107261184	-1.016013181	-1.316553526	-1.671316255	-1.66307228
9	0.302519497	1.114308738	1.574150529	1.642245571	3.47194548	3.628969412

10	2.031797709	1.190868114	2.442467517	3.154859084	4.616801859	6.263097135
11	1.916634945	0.806129128	3.346701123	2.048687386	4.046569213	6.281868308
12	2.75651032	0.700128361	4.313748707	1.729521529	2.733637692	4.445985376
13	1.618505127	0.957615565	5.700542411	2.243999798	2.72914859	2.716100313
14	0.275931448	0.943548007	4.983848134	2.48260806	2.237808196	0.236484004
15	-1.303132041	0.347867091	3.930405485	2.232771146	2.710786029	-1.571878981
16	-1.770407189	-0.41844745	0.788915566	0.630071196	0.879257248	-3.395937551
17	-1.262622632	-1.735211386	-1.437533482	-0.540890396	-0.42030921	-3.688884573
18	2.462531535	-1.596191927	-3.098583809	-1.696076925	-3.074384526	-0.878748402
19	10.62617156	0.723894332	-4.463546218	-2.361760769	-3.69296915	6.658226717
20	29.52098269	8.57135063	-4.4562113	-2.326389064	-2.175357406	24.96118626
21	61.24972521	24.5604943	-0.315199332	3.347094289	4.576591749	57.16271049
22	106.0914882	53.08344944	14.92700751	19.88841108	21.40264766	106.0971445
23	161.7226416	100.1415044	46.61618874	55.53091689	55.09039241	169.1578735
24	226.9342772	165.2133415	95.61500382	107.1672272	106.3652963	243.9904434
25	298.4198873	241.7949551	158.9651405	169.6225362	171.1299697	330.2026404
26	371.4763887	326.4812804	230.2474684	237.2404058	243.1100289	419.4451311
27	439.9018632	413.8905033	305.9864988	308.6545873	318.4311192	505.9696059
28	500.9153107	502.8652481	382.4033079	381.6605434	392.0634928	581.3565363
29	555.3201474	583.4976768	453.2470132	446.5441166	458.0263293	647.5723547
30	601.9208566	653.1747467	516.1116536	503.3215683	514.5175151	703.7264417
31	641.839018	710.7922816	566.4658602	548.653319	560.7804632	750.8339601
32	675.4598444	760.2068377	609.522167	587.2747053	598.1034338	789.7198184
33	703.0846943	798.768891	641.4168072	616.6648785	627.5927612	824.5520311
34	727.2508818	829.119848	667.6195341	642.0667362	652.7273646	854.7291825
35	747.5261416	852.8580853	684.651545	659.6246881	671.6242945	880.5645927
36	766.7314833	873.2315593	698.31103	673.4161958	687.0027448	899.6006423
37	780.9446559	888.2095367	706.6618666	682.4856335	699.4299646	917.4084715
38	794.345411	900.6354621	713.8764684	690.0573684	710.1632424	932.2107846
39	805.4534631	911.3235863	719.1120426	696.3495705	720.5132247	948.0158177

40	819.566977	924.7689758	725.1766061	699.9950843	728.2468758	961.0851311
**NTC**						
**Cycles**	**Fluorescence (ΔR)**					
	G7	G8	G9	G10	G11	G12
1	-27.17174347	-20.93617399	-14.58108194	-12.47148749	-8.707890754	-17.37602432
2	-21.69053407	-16.71219825	-5.995438108	-5.987416125	-3.668172066	-10.72552365
3	-19.47492467	-16.23782251	-3.100194275	-3.481744759	-2.363653379	-7.259022972
4	-18.24603527	-15.95096677	-0.845430442	-0.902153393	0.002625308	-3.433322297
5	-16.26760987	-16.25153503	0.143157391	0.496541973	0.834215996	-2.172581622
6	-13.73662126	-16.10709209	0.550414024	0.273842139	1.171221083	-1.352992946
7	-8.945212823	-15.19113171	0.588169217	0.290684065	0.90237145	-0.934626671
8	-3.991207773	-12.83186561	-0.164242118	-0.768844698	-0.386566246	-0.284735275
9	1.047400566	-9.229634867	0.051412954	-0.195739245	-0.6006925	0.131216664
10	4.135448884	-3.890150054	1.302648001	0.888618912	-1.003874078	-0.653314264
11	7.050305857	1.0453785	2.754612324	0.801754454	-1.229904431	-2.124729656
12	7.340412556	4.247566616	4.253838498	2.182896013	-0.458315605	-1.573618515
13	7.270930932	6.422295393	3.802662898	1.823394253	0.748227302	-0.75537896
14	5.604581587	6.644864215	3.370859312	3.209365032	2.041284861	1.720791584
15	5.146941035	6.471509178	1.152849775	1.596301656	1.838636166	2.781974009
16	4.811668696	5.028537378	0.58147345	1.732525956	1.754149152	3.491745008
17	4.742611759	4.852457454	-1.017817423	0.21880096	1.594153222	3.248236098
18	4.151271545	4.275832553	-1.293364562	0.324943641	1.442687846	2.943788921
19	3.308717755	4.272477031	-1.909745865	-0.374930003	0.477826797	2.036498109
20	4.111464596	3.703666389	-1.529545254	-0.912033376	-1.048007276	0.396450917
21	3.193028847	3.3364186	-1.018195092	-0.17778596	-1.048715088	0.089283721
22	2.859416706	3.096392357	-0.681298638	-0.156714332	-1.056592252	-0.297858751
23	0.698532769	2.322122993	-0.753063015	0.175992297	0.439121965	0.36557972
24	-0.510840805	1.666449315	-0.741450299	-0.171609232	0.434120588	0.023139325
25	-0.695366667	2.12764615	-0.794894332	-0.792945393	-0.770305632	-1.110360654

26	-1.284620913	1.235936225	-1.444674296	-0.805090111	-1.714759939	-1.403248324
27	-0.749851294	1.09701905	-3.226732795	-2.550143468	-2.447104831	-1.286225449
28	-1.671222578	-0.761920927	-4.154514188	-2.919940263	-1.485033459	-0.119098002
29	-0.85901727	0.185693086	-2.737895866	-2.561267474	-1.541656951	-0.859967533
30	-1.791324824	-0.449386664	-1.881542181	-2.181849455	-0.863136162	-0.972415544
31	-2.125819633	-0.639694368	-1.46836148	-0.852588474	-0.04132549	-0.940778647
32	-3.689654464	-2.657586416	-1.855868302	-0.729209855	0.756172014	0.645359535
33	-2.379794751	-1.952040923	-0.492147227	0.542960885	0.577464862	-0.628785044
34	-1.341064134	-2.067324791	0.061681923	-0.396286424	0.198654145	-0.164085918
35	0.418179573	-0.402087022	0.803778268	-0.348058918	-0.215418217	-1.123674604
36	-0.132699921	-0.944910799	0.621549667	0.655379941	0.92343638	0.37964789
37	-0.001501437	-0.973242558	1.292109516	0.047356033	-0.034175961	-0.509304944
38	-1.195911999	-2.340288221	1.948362065	1.108081843	0.699503703	0.215869394
39	-1.576899131	-3.283358746	3.084509498	0.576237608	-0.156635825	-0.057455407
40	-2.944672116	-4.417638806	4.746888868	4.333257173	1.630000484	0.855655836

File 5: Regression curve of RSPG1 gene amplification

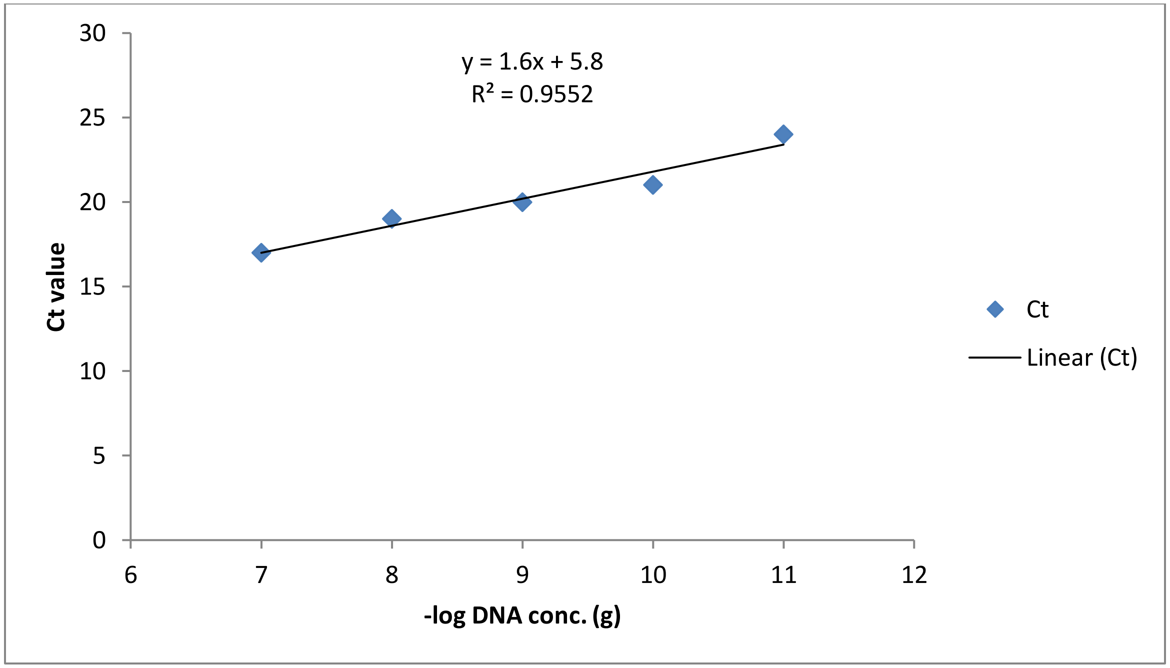

File 6: Regression curve of RSPG2 gene amplification

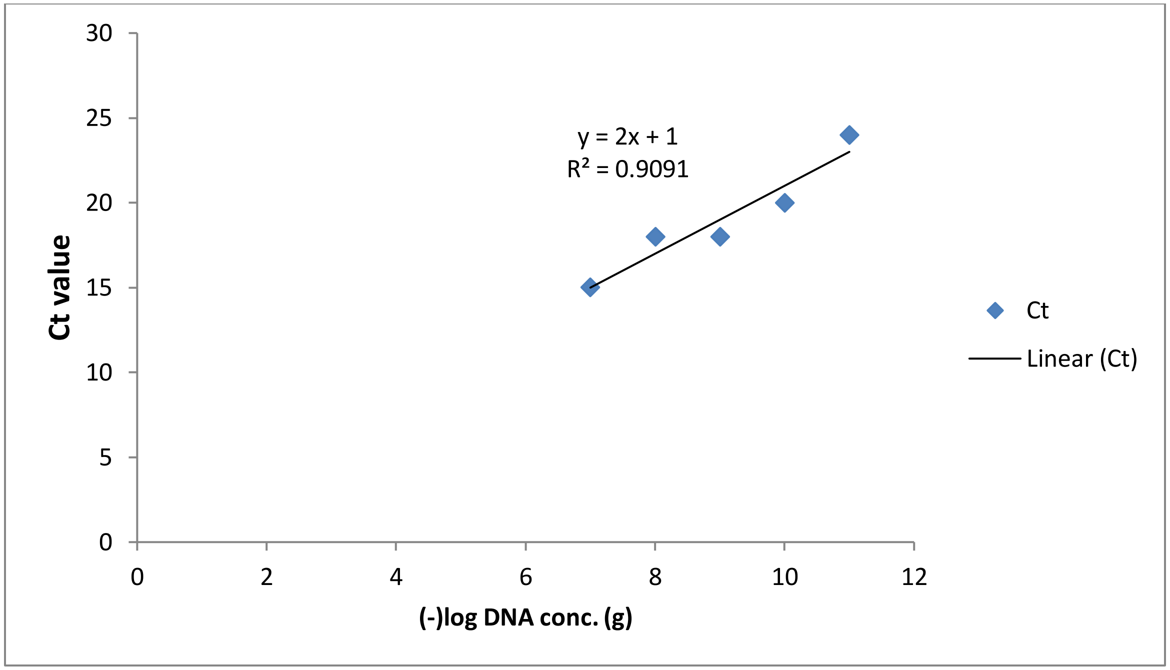

File 7: Regression curve of RSPG4 gene amplification

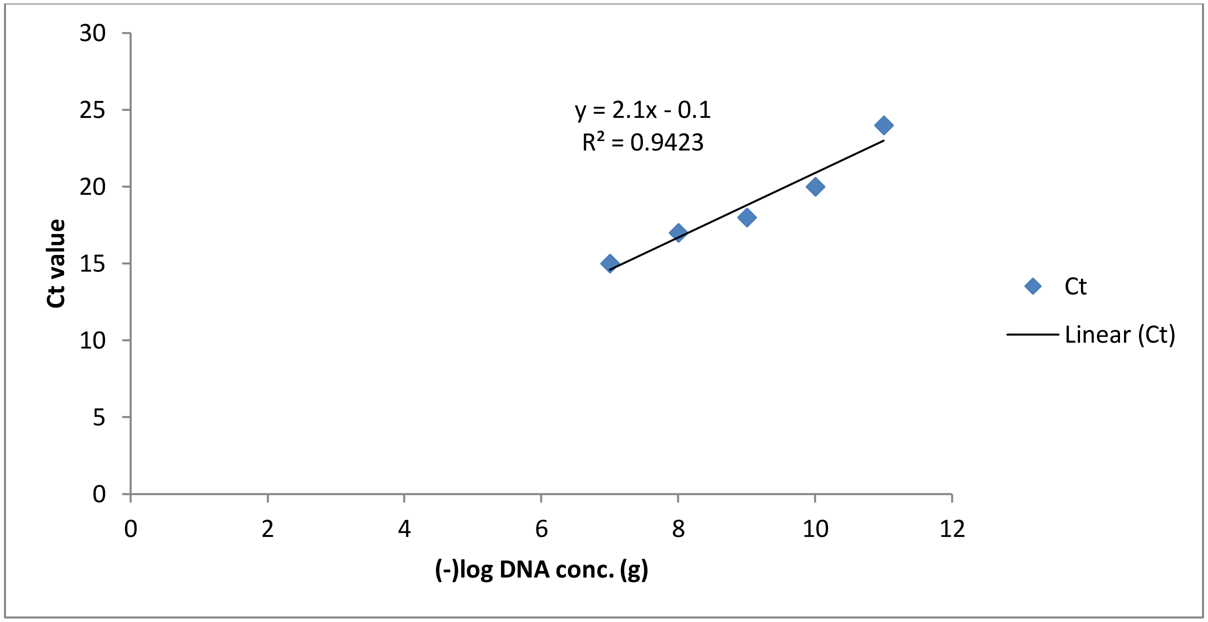

File 8: Regression curve of RSPG5 gene amplification

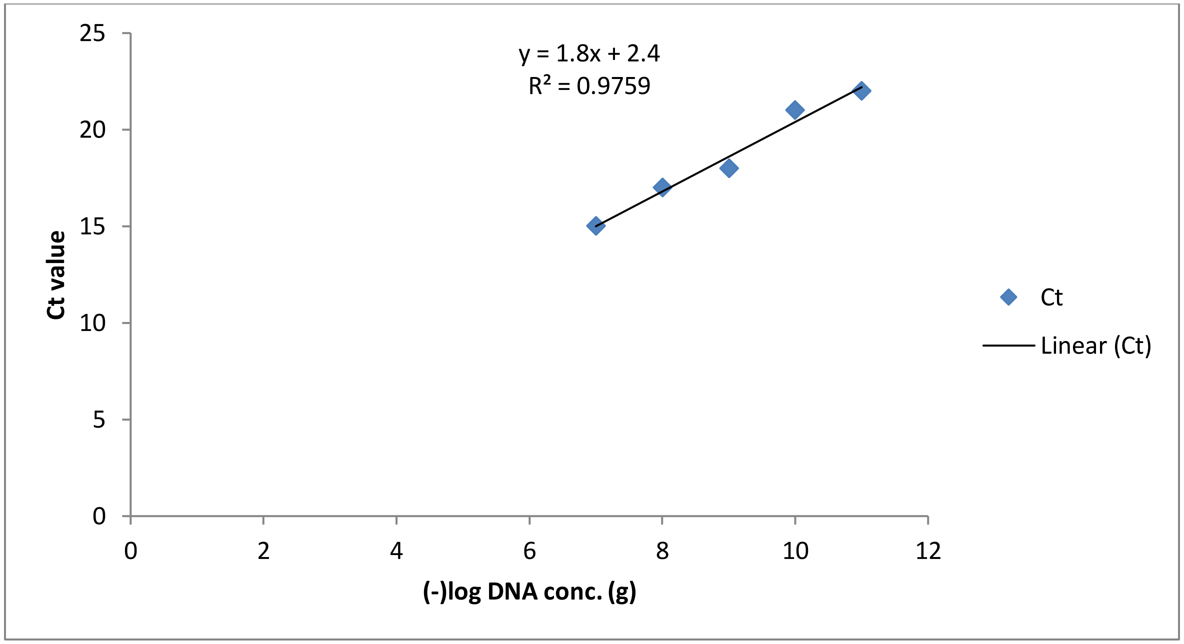

Supplementary Figure 1: Melt curves of the RSPG q-PCR primer sets.

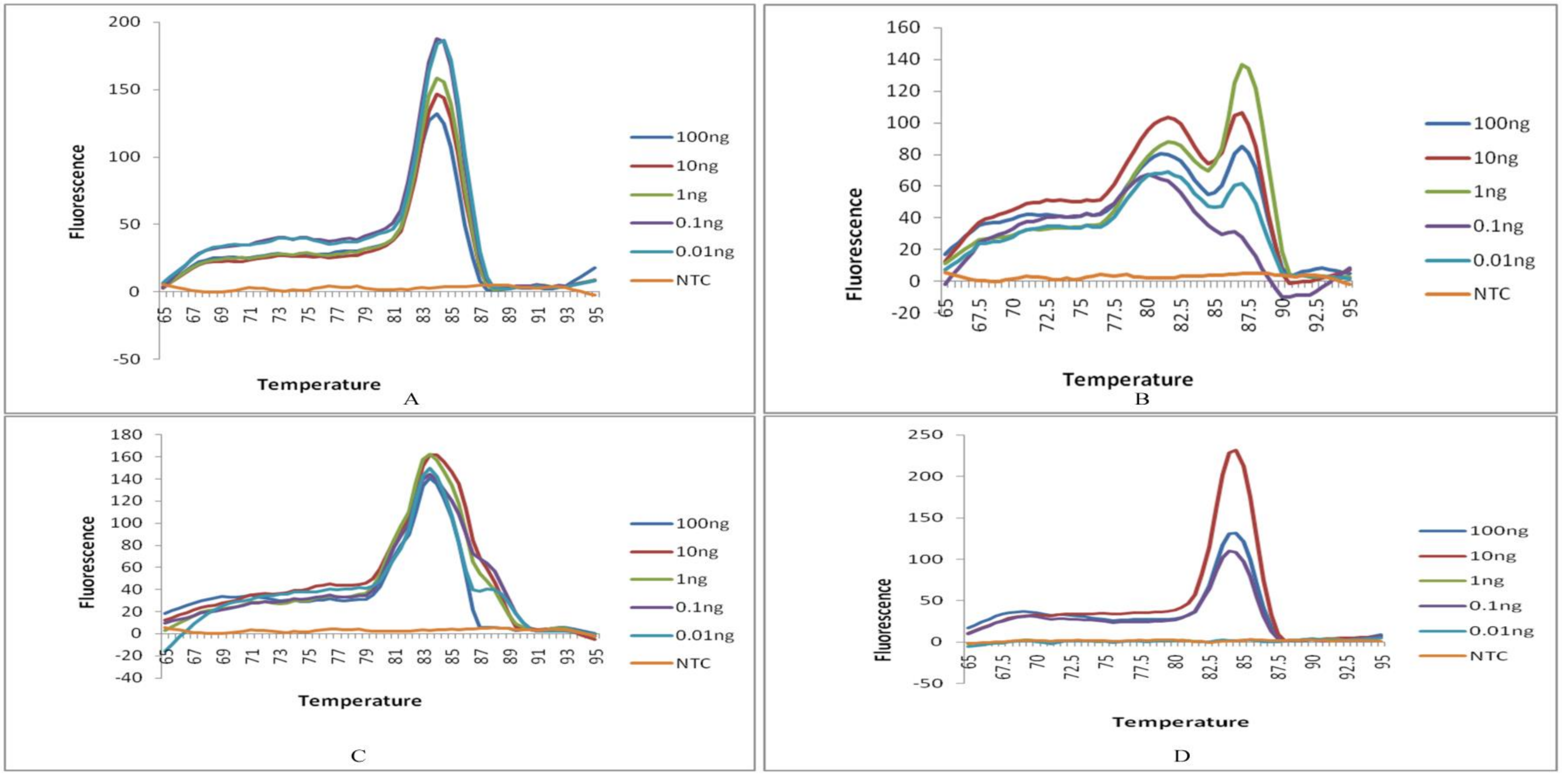

Supplementary Figure 2: LAMP primers spanning the gene region used in the study

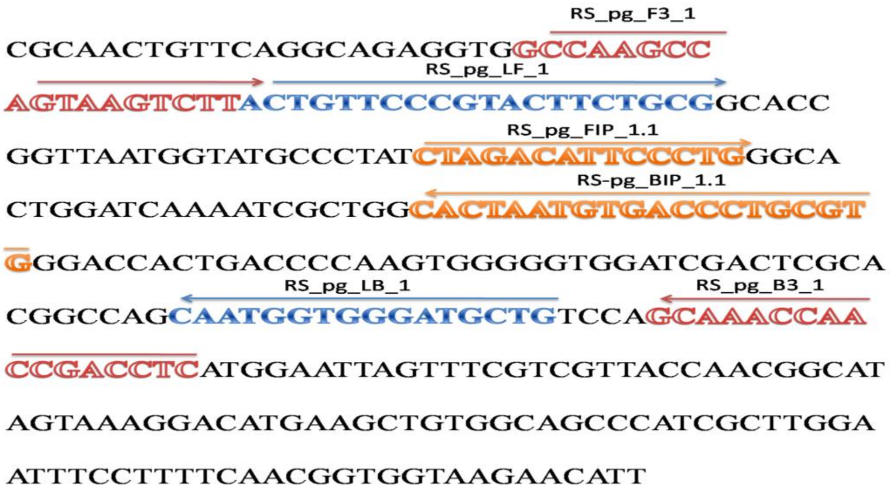

Supplementary Figure 3: Raw image file of Figure 4 (a) *R. solani* AG-1 IA amplification

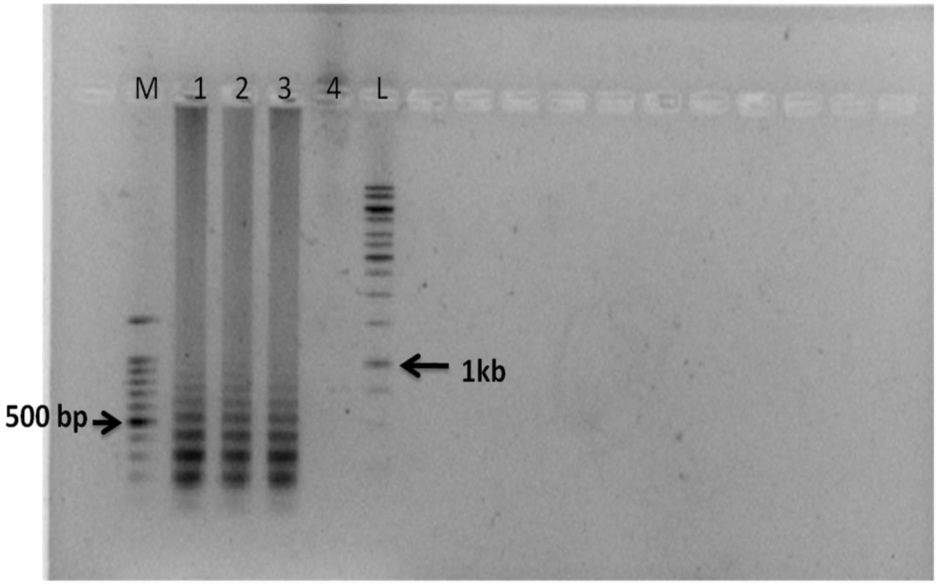

M: 100bp ladder (Promega); 1: *R. solani* AG-1 IA PURS1; 2: *R. solani* AG-1 IA PURS2 3: *R. solani* AG-1 IA PURS3; 4: No Template control; L: 1kb (Generuler). (B) Specificity assay of the LAMP assay. L: 1kb (Generuler)

Supplementary Figure 4: Raw image file of Figure 4 (b) *R. solani* AG-1 IA amplification

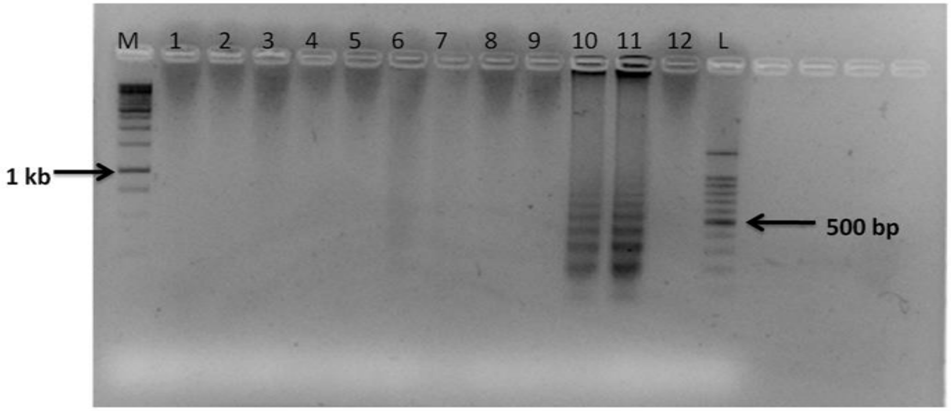

L: 1kb (Generuler) ; 1: *Colletotrichum capsici*; 2: *Sclerotium rolfsii*; 3: *Trichoderma asperellum;* 4: *Fusarium oxysporum*; 5: *Alternaria alternata*; 6: *Ustilaginoidea virens*; 7: *Curvularia prasadii*; 8: *Cochiliobolus tuberculatus*; 9: *Pseudomonas plecoglossicida* 10: *R. solani* PURS1; 11: *R. solani* PURS2; 12: No Template control; M: 100 bp (Promega). (C) M: 100 bp (Promega)

Supplementary Figure 5: Raw image file of Figure 7 (a) *R. solani* AG-1 IA amplification

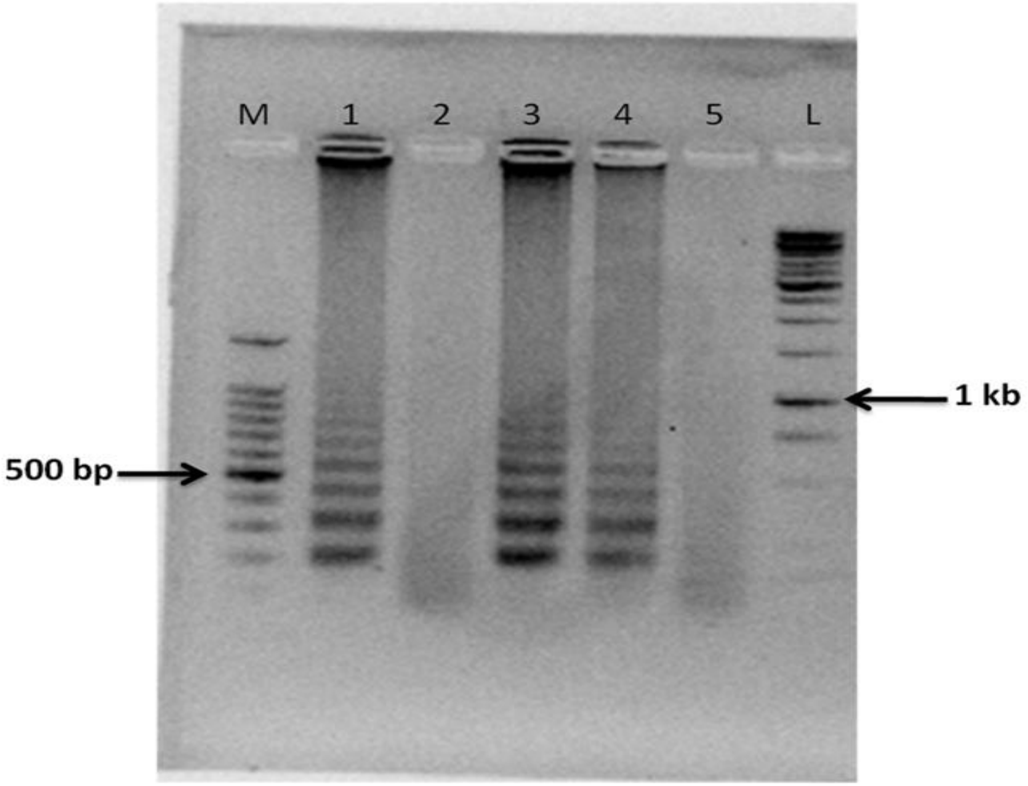

M: 100 bp Marker (Promega); 1: *R. solani* AG-1 IA PURS2; 2: healthy plant; 3: Infected plant; 4: Soil DNA from rice field; 5: NTC; L: 1kb marker (Generuler)
